# Quantitative Proteomics Reveals the Dynamic Regulation of the Tomato Proteome in Response to *Phytophthora infestans*

**DOI:** 10.3390/ijms22084174

**Published:** 2021-04-17

**Authors:** Kai-Ting Fan, Yang Hsu, Ching-Fang Yeh, Chi-Hsin Chang, Wei-Hung Chang, Yet-Ran Chen

**Affiliations:** 1 Agricultural Biotechnology Research Center, Academia Sinica, Taipei 11529, Taiwan; kaitingfan@sinica.edu.tw (K.-T.F.); sheuyoung@gmail.com (Y.H.); cfyeh@gate.sinica.edu.tw (C.-F.Y.); as0190450@gate.sinica.edu.tw (C.-H.C.); whchang@gate.sinica.edu.tw (W.-H.C.); 2 Molecular and Biological Agricultural Sciences Program, Taiwan International Graduate Program, Academia Sinica, Taipei 11529, Taiwan; 3 Graduate Institute of Biotechnology, National Chung Hsing University, Taichung 402, Taiwan

**Keywords:** plant pathogenesis responses, quantitative proteomics, data-independent acquisition, *Phytophthora infestans*

## Abstract

Late blight (LB) disease is a major threat to potato and tomato production. It is caused by the hemibiotrophic pathogen, *Phytophthora infestans*. *P. infestans* can destroy all of the major organs in plants of susceptible crops and result in a total loss of productivity. At the early pathogenesis stage, this hemibiotrophic oomycete pathogen causes an asymptomatic biotrophic infection in hosts, which then progresses to a necrotrophic phase at the later infection stage. In this study, to examine how the tomato proteome is regulated by *P. infestans* at different stages of pathogenesis, a data-independent acquisition (DIA) proteomics approach was used to trace the dynamics of the protein regulation. A comprehensive picture of the regulation of tomato proteins functioning in the immunity, signaling, defense, and metabolism pathways at different stages of *P. infestans* infection is revealed. Among the regulated proteins, several involved in mediating plant defense responses were found to be differentially regulated at the transcriptional or translational levels across different pathogenesis phases. This study increases understanding of the pathogenesis of *P. infestans* in tomato and also identifies key transcriptional and translational events possibly targeted by the pathogen during different phases of its life cycle, thus providing novel insights for developing a new strategy towards better control of LB disease in tomato.

## 1. Introduction

Late blight (LB), caused by the oomycete *Phytophthora infestans*, is one of the most notorious plant diseases to afflict solanaceous plants [1]. LB is difficult to control and causes severe losses of up to 100% in the production of potato and tomato crops [2]. LB was the major factor contributing to severe crop loss in Ireland in early 1840, causing the Great Famine and resulting in the population of the area falling by 20–25%. Until now, this disease remains one of the biggest threats to tomato and potato production. In the United States, LB has caused up to 7% yield losses in tomato crops and approximately 3.5% yield losses in potato crops over the last two decades [2]. The worldwide economic losses due to LB and the cost of controlling this disease are estimated to exceed $5 billion in tomato crops [3] and about $7 billion in potato crops [2] annually.

*P. infestans* destroys all the leaves, roots, tubers, and fruit of susceptible plants [4]. After infection of susceptible hosts by this pathogen, local lesions pervade the whole plant within a few days, eventually causing the death of the plant. The sporangia produced on an infected plant organ can be effectively dispersed by wind or by splashes from raindrops [5]. *P. infestans* exhibits a two-phase life cycle; it is initially hemibiotrophic, showing an asymptomatic biotrophic phase of infection. This is followed by a necrotrophic phase that is characterized by the degradation of the host tissue [6]. During the biotrophic stage, *P. infestans* forms appressoria, primary and secondary hyphae and a specialized structure called haustoria within living plant cells and obtains nutrients without killing the plant [7]. In this stage of pathogenesis, *P. infestans* suppresses the plant immune system and the programmed cell death (PCD) responses that limit biotrophic infection. At the necrotrophic stage, the pathogen hyphae ramify throughout the plant and toxins from the pathogen are injected into the host cells causing necrosis of the infected tissue [8].

To gain a comprehensive insight into the dynamic plant molecular mechanisms that respond to the hemibiotrophic infection of *P. infestans*, several time-course quantitative transcriptomics studies have been performed on tomato or potato infected by this pathogen [9,10,11]. In potato, massive transcriptional reprogramming was observed at the end of the early biotrophic infection stage but the pathogenesis-related (PR) and hypersensitive response (HR) genes were mainly upregulated at the later necrotrophic stage [9]. Using microarray analysis, genes encoding transcription factors and components of signaling pathways were shown to be upregulated by *P. infestans* in a resistant tomato cultivar, but suppressed in a susceptible one [10]. Due to the accuracy of the microarray technique, five genes considered to be involved in plant resistance in the microarray-based transcriptomics study were validated by real-time quantitative reverse transcription polymerase chain reaction (qRT-PCR) analysis: endochitinase 3 precursor, glyceraldehyde 3-phosphate dehydrogenase B, probable GST, carbonic anhydrase, and basic glucanase. More recently, a more accurate transcriptomics analysis using the next-generation sequencing technique was performed to observe the transcriptomic change in tomato from the well-defined biotrophic to the necrotrophic pathogenesis stages of *P. infestans* infection [11].

Furthermore, proteomics was used to investigate the pathogenic regulation dynamics of the plant proteome by this pathogen. The interaction of potatoes and *P. infestans* was examined by quantifying the changes in the potato proteome after infection, both in a temporal manner and between the resistant and susceptible cultivars [12,13,14]. These studies provided further direct evidence about the production of key proteins that can directly interact with this pathogen and proceed with the metabolic reaction for the defense metabolites or energy homeostasis. However, to date, there has been no comprehensive study reporting the dynamics of the tomato proteome in response to *P. infestans*. In a previous proteomic study that used two-dimensional electrophoresis (2-DE) and matrix-assisted laser desorption/ionization time-of-flight/time-of-flight (MALDI-TOF/TOF) for protein identification, only 56 proteins were identified in the proteome of resistant and susceptible tomato cultivars in response to *P. infestans* infection [15].

In this study, a data-independent acquisition (DIA) proteomics approach was applied to reveal the dynamics of the proteome change in tomato from the early (biotrophic phase) to the late stage (necrotrophic phase) of *P. infestans* infection. By establishing a DIA spectra library from two-dimensional-liquid chromatography with tandem mass spectrometry (LC-MS/MS) analysis operated in the data-dependent acquisition (DDA) mode, more proteins were identified compared to the use of a spectral library established by single-dimensional LC-MS/MS. This approach enabled the profiling of the tomato proteome in a high-throughput manner, and can potentially be used to facilitate the analysis of the dynamics of different pathogenic responses in plants.

## 2. Results and Discussion

### 2.1. P. infestans Pathogenesis Assessment in Tomato Leaves

**Figure 1:**
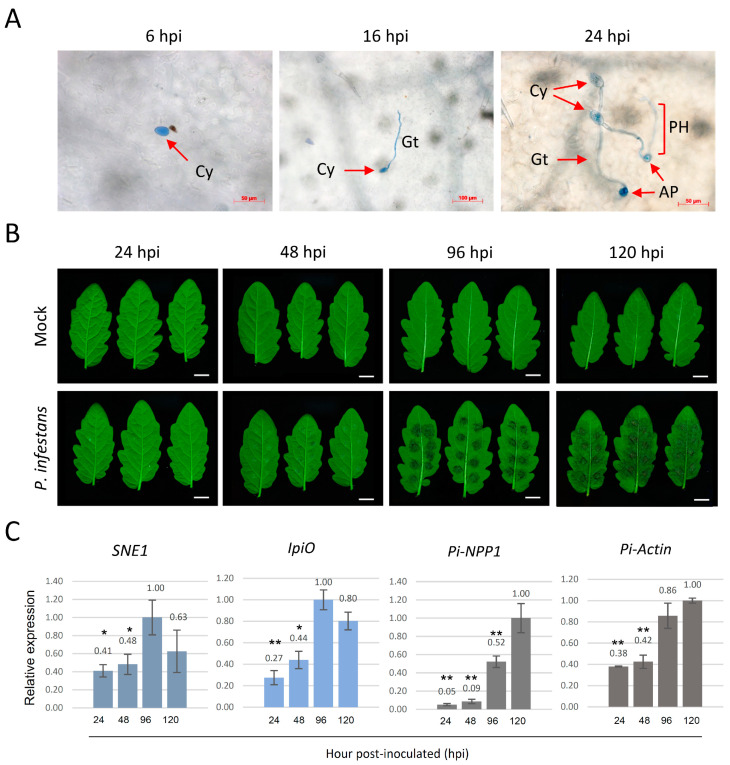
Inoculation of detached tomato leaflets by *P. infestans.* Detached leaflets of five-week-old tomato plants were inoculated with *P. infestans* by placing a 20 μL drop of inoculum at eight spots on the abaxial surface of the leaflets. (**A**) Microscopic assessment of *P. infestans* developing on tomato leaflets at different time points post-inoculation after staining with trypan blue. At 6-h post-inoculation (hpi), the pathogens remained as cysts and started to germinate at 16 hpi. At 24 hpi, the primary hyphae (PH) were developed and the appressorium (Ap) structure was generated, indicating that the pathogen was ready to invade the plant cells. (**B**) Example leaflets showing *P. infestans* lesion development at 24, 48, 96 and 120 hpi. The white bar represents 1 cm. (**C**) Bar graphs showing the relative band intensity of reverse transcription-polymerase chain reaction (RT-PCR) products of *P. infestans* marker genes, *SNE1*, *IpiO*, and *PiNPP1.1* is shown in Figure S1. The RNA samples were extracted from tomato leaflets at 24, 48, 96, and 120 hpi. The relative transcript abundance is shown as a proportion of the most intense band in each gel image. The expression level of *P. infestans* actin was used to evaluate the biomass of the pathogen in tomato leaves. The expression levels of biotrophic stage markers (*SNE1* and *IpiO*) were reduced after 96 hpi, and the necrotrophic stage marker (*PiNPP1.1*) was continuously increased from 24 hpi to 120 hpi. The error bars are standard deviations and the graph represents the combined data from three biological replicates (n = 3). Differentially regulated genes with a *p*-value of less than 0.05 or a *p*-value of less than 0.01 are marked with single or double asterisks, respectively. Cy, cyst; Gt, germ tube; AP, appressorium; PH, primary hyphae.

A time-series phenotypic assessment of the leaf from a susceptible tomato (*Solanum lycopersicum*) cultivar (CL5915) challenged with *P. infestans* was subjected to microscopic and macroscopic examination (Figure 1A,B). Leaflets detached from 35-d-old tomato plants were inoculated with droplets of *P. infestans* sporangia suspension and collected at 6, 16, 24, 48, 96, and 120-h post-inoculation (hpi). Three biological replicates were used at each time point in the phenotypic assessment. Because there was no obvious disease phenotype at the early time points, the pathogen development on leaflets at 6, 16, and 24 hpi were stained with trypan blue. After the staining, the growth phenotype of the pathogen was recorded by picking thirty *P. infestans* spores randomly. Microscopy showed that 20% (6/30) sporangia started to germinate and 80% (24/30) sporangia remained at the cyst stage at 6 hpi (Figure 1A). At 16 hpi, about 90% of the resting cysts of *P. infestans* germinated and developed geminating tubes, and only ~3% (1/30) of the sporangia remained as cysts. The appressorium structures were first observed at 16 hpi and accounted for only ~7% (2/30) of the total cysts. At 24 hpi, appressoria appeared in ~73% (22/30) of the observed cysts, and primary hyphae could be observed extending through the host tissue. No disease symptoms on the leaf were observed macroscopically at 24 hpi and only little inconspicuous black spots were discovered at 48 hpi (Figure 1B). Water-soaked lesions were not obvious until 96 hpi and the average area of the lesions observed at this time point was 0.71 ± 0.12 cm^2^. At 120 hpi, the lesion area had covered over 80% of the leaf surfaces and aerial mycelium were visually observable surrounding the center of the droplets, indicating a well-established necrotrophic phase.

To better assess the pathogenesis phase at the molecular level, the expressions of *P. infestans* genes that mark different pathogenesis stages were analyzed by reverse transcription-polymerase chain reaction (RT-PCR) in the leaflets at 24, 48, 96, and 120 hpi (Figure 1C and Figure S1). Two *P. infestans* RXLR effector genes *SNE1* and *IpiO* (also named *Avr-blb1*) were used as marker genes for infection at the biotrophic stage. *SNE1* has been reported to be specifically expressed at the transcriptional level during the biotrophic stage in infected tomato [16], and *IpiO* has been detected in the early stage of infection, but not in sporangia or old mycelia [17]. Another gene *PiNPP1.1* which encodes a cell death-inducing protein and thus marks the necrotrophic stage of infection [18] was used in this study. The expression of *SEN1* and *IpiO* was detected from the early stage of infection (24 hpi) and continually accumulated during the biotrophic phase until 96 hpi. The expression of both biotrophic markers decreased at 120 hpi, suggesting that the pathogen transited from biotrophy to necrotrophy at 96 hpi. In contrast, *PiNPP1.1* expression was detected at low abundance until 96 hpi and was mainly expressed at the later stages of the interaction. The expression of *P. infestans* actin was used to evaluate pathogen growth. Expression of this gene was continuously increased from 24 hpi to 120 hpi. Taken together, *P. infestans* exhibited a biotrophic invasion in tomato cv. CL5915 at 24 hpi by initiating the penetration. The time points 48 and 96 hpi represented the transition phase (the change from biotrophic to necrotrophic growth), and the necrotrophic phase, respectively.

### 2.2. Proteomics Analysis and Quantification Using the DIA Approach

**Figure 2:**
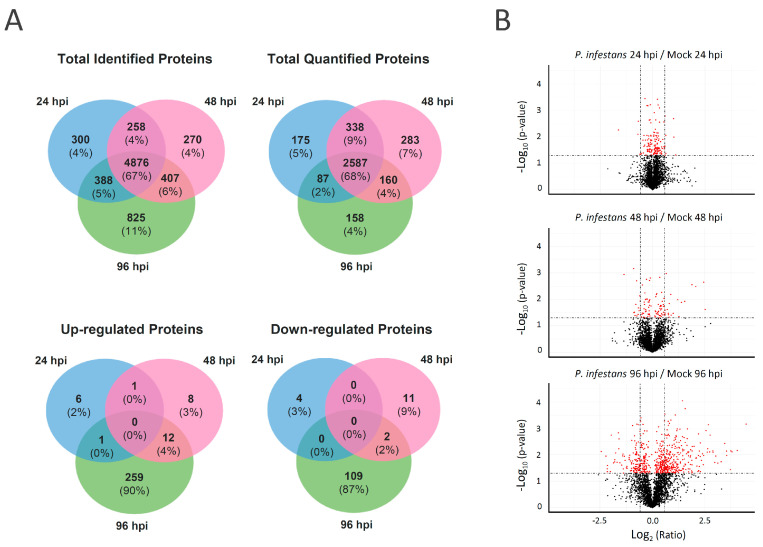
Proteome changes in tomato leaf between 24, 48 and 96 h post-inoculation with *P. infestans* compared to post-mock-inoculation at 24, 48, and 96 h, respectively**.** (**A**) Venn diagrams showing the unique and shared tomato or *P. infestans* proteins identified, quantified in three biological replicates, or showing a significant difference in abundance with a fold change greater than 1.5 or less than 0.67 in quantity (*p* < 0.05) due to inoculation. (**B**) Volcano plots showing the protein abundance ratio of *P. infestans*-inoculated over the mock group at 24, 48 and 96 hpi. Following liquid chromatography-mass spectrometry (LC-MS) analysis and data-independent acquisition (DIA) quantification, *t*-test-based significance values (log_10_ (*p*-value)) were plotted versus log_2_ (protein quantity ratio for all proteins between infected and mock). Differentially regulated proteins with *p* < 0.05 are plotted in red. A level of protein abundance change of 1.5 or 0.67-fold is marked with a dashed line.

To study the plant pathogenesis responses at the protein level across the early biotrophic to transition and later necrotrophic phases, tomato leaflets at 24, 48, and 96 hpi with *P. infestans* were subjected to proteomic analysis using data-independent acquisition (DIA). A DIA spectral library containing 11,563 proteins and 65,763 peptide transition groups from tomato and *P. infestans* was constructed by the analysis of the pooled tryptic peptide samples with or without fractionation by high-pH reversed-phase liquid chromatography (RPLC) using LC-MS/MS operated in data-dependent acquisition (DDA) mode. A total of 7324 proteins, 6631 tomato proteins, 678 *P. infestans* proteins (with at least 1 unique peptide), and 15 protein groups shared by tomato and *P. infestans* were identified in the DIA proteomics analysis of the time-series experiments. About 67% of tomato proteins were identified in samples collected at all time points (Supplemental Tables S1 and S2) and ~4–11% of the proteins were exclusively identified at a single time point (Figure 2A). To identify the tomato proteins regulated by this pathogen, the abundance of each protein in the plants inoculated with the pathogen was compared to the abundance in the mock-treated plants at the same time point. An average fold change of protein abundance in the pathogen and mock-treated plants from three biological replicates of ≥1.5 or ≤0.67 with significance (*p* < 0.05) was considered as up- or downregulated by *P. infestans*, respectively (Figure 2B). In all, 3165, 3345 and 2973 tomato proteins were quantified at 24, 48, and 96 hpi, respectively. In the quantitative analysis, 8, 21, and 270 tomato proteins were determined to be upregulated at 24, 48 and 96 hpi, respectively. In addition, 4, 13, and 110 tomato proteins were found to be downregulated at 24, 48 and 96 hpi, respectively. No protein was found to have differential up- or downregulation at all the time points and only ~4% of upregulated and ~2% of downregulated proteins were identified at both 48 and 96 hpi. The data, therefore, showed that the tomato proteome was barely changed during the biotrophic stage of infection at 24 hpi; in contrast, only certain proteins were quantified as regulated at the transition stage at 48 hpi; and more than 10% of the quantified proteins were regulated at the late necrotrophic stage at 96 hpi.

### 2.3. Functional Classification of the Proteins Regulated by P. infestans

**Figure 3:**
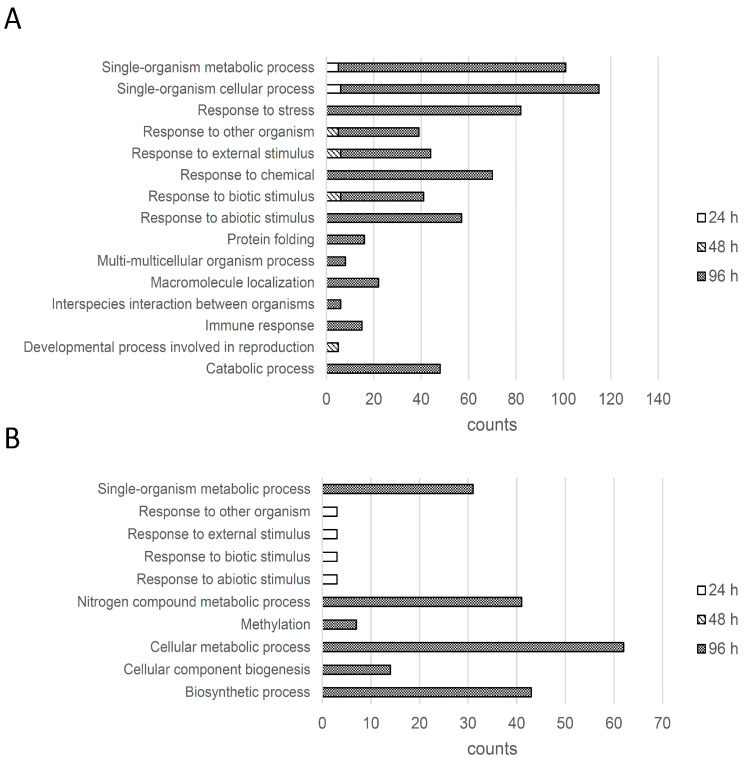
Functional analysis of biological processes for tomato proteins (**A**) upregulated or (**B**) downregulated at 24, 48, or 96 h post-inoculation with *P. infestans*. Tomato proteins up- or downregulated by *P. infestans* were matched to the Arabidopsis homolog proteins by Protein Basic Local Alignment Search Tool (BLASTP) against The Arabidopsis Information Resource (TAIR) database. The counts of these Arabidopsis homolog proteins were compared at different time points in the enriched function group with Gene Ontology (GO) categorization using the Database for Annotation, Visualization and Integrated Discovery (DAVID) v.6.8 (*p* < 0.05).

To understand the implications of the differentially regulated proteins in this study, the quantified proteins were subjected to functional categorization analysis using Gene Ontology (GO). In this analysis, we used The Arabidopsis Information Resource (TAIR) database combined with Protein Basic Local Alignment Search Tool (BLASTP) to identify the protein functions that showed a significant change in the *P. infestans*-treated and mock-treated samples. The upregulated and downregulated proteins were classified into 15 and 10 enriched categories, respectively (Figure 3). At 24 hpi, the enriched functional categories for the upregulated proteins were “single-organism metabolic process” and “single-organism cellular process”; the downregulated proteins were “response to other organism”, “response to external stimulus”, “response to biotic stimulus”, and “response to abiotic stimulus”. At 48 hpi, the enriched functional categories for the upregulated proteins were “developmental process involved in reproduction”, “response to other organism”, “response to external stimulus” and “response to biotic stimulus” but there were no enriched categories for the downregulated proteins. At 96 hpi, the enriched functional categories for the upregulated proteins were “catabolic process”, “immune response”, “interspecies interaction between organisms”, “macromolecule localization”, “multi-multicellular organism process”, “protein folding”, “response to abiotic stimulus”, “response to biotic stimulus”, “response to chemical”, “response to external stimulus”, “response to other organism”, “response to stress”, “single-organism cellular process” and “single-organism metabolic process”, and the enriched functional categories for the downregulated proteins were “biosynthetic process”, “cellular component biogenesis”, “cellular metabolic process”, “methylation”, “nitrogen compound metabolic process” and “single-organism metabolic process”.

The upregulated proteins that are pathogen resistance-associated fall into enriched function categories like “immune response”, “interspecies interaction between organisms”, “response to biotic and abiotic stimulus”, “response to external stimulus”, “response to other organism”, and “response to stress”, suggesting that these functions in the plant were positively regulated during the tomato-*P. infestans* interaction. Conversely, proteins related to the metabolic pathways or cellular biosynthesis processes were mostly downregulated, suggesting that metabolic flux may be altered by the attack of this pathogen.

### 2.4. Changes Associated with Direct Defense

Tomato proteins involved in different pathogen-prohibiting functions were found to be regulated by *P. infestans* (Table 1 and Table S1). Three tomato beta-glucosidases (Solyc01g008620, Solyc10g079860, and a protein group of Solyc01g059965 and Solyc01g060020) for hydrolyzing the major constitutes (1→3)-β-d-glucans, (1→6)-β-d-glucans and cellulose of the oomycete cell wall [19] were observed to be regulated by *P. infestans*. Among these beta-glucosidases, the expression of one of the protein groups (Solyc01g059965 and Solyc01g060020) that encodes a putative beta-1,3-endoglucanase sequence was downregulated with a fold change of 0.62 at 24 hpi, but upregulated with a fold change of 3.67 at 96 hpi. Two other quantified beta-glucosidases Solyc01g008620 and Solyc10g079860 did not have a significant change in abundance at 24 hpi, but were increased by 5.91 and 16.62-fold, respectively, at 96 hpi. Four proteins with chitinase activity (Solyc10g055800, Solyc04g072000, Solyc10g055810, Solyc02g082920) for hydrolyzing the chitin of the fungal cell wall were all found to be upregulated only at 96 hpi with a fold change from 3.52 to 7.90. PR-5 protein (Solyc08g080670), which can bind to the mannose phosphate groups of the fungal cell walls and exhibit a broad spectrum in fungal resistance [20], was downregulated with a fold change of 0.32 at 24 hpi but was not regulated at later time points. The PR-5 family proteins (also called thaumatin-like proteins) which include the closely related proteins permatin, osmotin and zeamatin, may cause fungal hyphae leak and rupture likely via disrupting the fungal membrane permeabilization [21] and overexpressing PR-5 causes enhanced resistance against necrotrophic fungus in multiple plant species [22]. In addition, several proteins that may have roles in defense but for which direct studies to examine their biological functions are lacking were also found to be regulated. One PR-10 family protein named norcoclaurine synthase (NCS; Solyc07g005370) was identified to have a fold change of 0.59 at 96 hpi. This protein belongs to the Bet v 1 family which have RNase activity [23,24] and cysteine protease inhibition activities [25], and participate in anti-bacterial, anti-fungal, anti-viral, and anti-nematode activity. However, three other PR-10 family proteins with accession numbers Solyc09g090990, Solyc09g091000, and Solyc09g090980 showed a 6.26, 10.98, and 22.30-fold increase at 96 hpi, respectively. Two of them Solyc09g090990 and Solyc09g090980 were also, respectively, 5.45 and 2.31-fold upregulated at 48 hpi. PR-4 (Solyc01g09724) and PR-4b (Solyc01g097280) proteins which are bifunctional enzymes with both RNase and DNase activity and involved in regulating HR [26] were also shown to be increased at 96 hpi by 2.14 to 3.0-fold.

Of the defense proteins identified to be regulated by *P. infestans*, the beta-glucosidases and PR-5 protein involved in the disruption and blocking of the pathogen cell wall were repressed at the early stage of the pathogenesis. This repression may facilitate the early development of the pathogen, and establish successful penetration into plant cells at the biotrophic phase. In the *P. infestans*-resistant potato cultivar, endo-1,3-beta-glucosidase was upregulated as early as 6 hpi but not in the susceptible potato cultivar [13]. Furthermore, the previously claimed resistant potato cultivar became susceptible to another *P. infestans* isolate that caused severe lesion areas after inoculation had upregulated endo-1,3-beta-glucosidase at a later time point [14]. Therefore, the early induction of these hydrolytic enzymes in the resistance cultivar could be the key to plant resistance to this pathogen. In our study, tomato beta-glucosidases were not regulated by *P. infestans* at the early time point, which may be related to the plant susceptibility for this pathogen. However, at the later time point after *P. infestans*-inoculation, tomato proteins related to the disruption of the glucans were highly expressed. This may reflect enhanced plant resistance by the degradation of the hyphal cell wall of the oomycete, which not only renders it susceptible to cell lysis but also releases beta-1,3-glucans which serve as elicitors [27] to initiate a wide range of localized and systemic defense responses. Moreover, the proteins involved in regulating HR were induced at 96 hpi; this response may contribute to the triggering of rapid cell death in the local region surrounding the infection area to prevent the spread of the pathogen.

### 2.5. Changes Associated with Immune Regulation

Several tomato proteins involved in regulating immune responses were identified to have an abundance change in response to the pathogenesis of *P. infestans* (Table 2 and Table S1). Three PR-1 family proteins (Solyc00g174340, Solyc09g007010, and Solyc01g106620) were upregulated at 96 hpi with fold-changes from 4.91 to 7.47. The protein encoded by the gene Solyc00g174340, named pathogenesis-related protein 1b (PR-1b), is the precursor of the peptide hormone, cysteine-rich secretory protein, antigen 5 and PR-1 (CAP)-derived peptide 1 (CAPE1). PR-1b was upregulated at 48 and 96 hpi with a fold change of 1.51 and 7.47, respectively. CAPE1 derived from PR-1b has been demonstrated to activate tomato antiherbivore and antipathogen responses via jasmonic acid (JA) and salicylic acid (SA)-regulated responses [28]. The production of CAPE1 has been suggested to balance excessive JA production and facilitate the biosynthesis of SA in plants [29]. On the other hand, the sterol-binding activity of tobacco PR-1b contributes to the depletion of the lipids required for oomycete *Phytophthora brassicae* to inhibit the growth of this pathogen [30]. Several proteins involved in the effector-triggered immunity (ETI)-triggered programmed cell death (PCD) were all upregulated at 96 hpi, including phytophthora-inhibited protease 1 (PIP1; Solyc02g077040) with a 3.0-fold change, hypersensitive-induced response protein (HIR; Solyc06g071050) with 1.61-fold change and response to desiccation 21a (RD21a; Solyc04g078540) with 1.66-fold change. PIP1 is a tomato apoplastic cysteine protease that triggers cell death and fungal resistance [31]. The enzyme activity of PIP1 has been shown to be inhibited by an effector EPIC2B produced from *P. infestans* [32]. The function of HIR protein is associated with HR and cell death, positively contributing to the inhibition of hemibiotrophs [33,34]. HIR proteins have been shown to help the recognition of the *Pseudomonas syringae* effector AvrRpt2 by interacting with the R protein *Pseudomonas syringae* 2 (RPS2) via increasing the local concentration of RPS2 at plasma membrane microdomains [34]. RD21a contributes to the resistance to necrotrophic fungi in Arabidopsis and functions as a PCD-promoting protease which is released from the ER body or vacuoles to the cytoplasm [35]. This protein has also been shown to control stomata closure and its degradation by ubiquitin E3 ligase SINAT4 was recently found to be triggered by the bacterial type III effector AvrRxo1 [36]. 

Among all the quantified proteins, carbonic anhydrase (CA; Solyc02g067750) which is essential for various biological processes including stomatal aperture, respiration, pH regulation, and CO_2_ homeostasis in plants [37] was upregulated with the greatest fold change at 96 hpi (~320-fold change). This protein is involved in attenuating flg22-triggered immunity [38], and functions in the *Pto:avrPto* mediated-HR in plant-*P. synrigae* interaction [39]. The gene expression of *CA* was more suppressed in the compatible interaction than the incompatible interaction between *P. infestans* and potato (*Solanum tuberosum*) [40]. Silencing this plastic *CA* gene results in higher susceptibility to *P. infestans* in tobacco (*Nicotiana benthamiana*) than the wild type [40]. 

Being a more general immune regulator, enhanced disease susceptibility 1 (EDS1; Solyc06g071280) was 1.91-fold upregulated at 96 hpi. EDS1 functions as a SA regulator and key ETI mediator for several toll-interleukin-1 receptor (TIR) NB-LRR resistance proteins against virulent pathogens in different plant species [41,42,43,44,45]. This protein enhances the SA-dependent defense response against the oomycete *Phytophthora parasitica* [44,46]. It has been proposed that EDS1 and SA signaling pathways are operated in parallel in defense responses. The EDS1-phytoalexin deficient4 (PAD4) signaling is distinct from the known SA-compensatory route involving MAPK signaling and can regulate both SA-dependent and SA-independent gene expression sectors [47]. In contrast to proteins initiating the ETI-triggered PCD, Kunitz-type protease inhibitor (KTI; Solyc03g098730) which antagonizes the pathogen-associated PCD [48] was 1.91-fold upregulated at 96 hpi of *P. infestans*. The expression of *KTI* is induced by SA and some PCD-eliciting toxins from necrotrophic fungi [48] and knocking out the *KTI* gene causes more H_2_O_2_ accumulation in plants [49].

In summary, *P. infestans* regulates the PR-1 protein family for JA and SA immune signaling and defense responses at 48 and 96 hpi. After 96 h of pathogen inoculation, CA protein, which functions to suppress the PTI responses and the activation HR development, was highly induced. In the meantime, the induction of EDS1 for more general deference responses may further counteract the immune suppression of pathogen effectors. At 96 hpi, we also observed upregulated proteins that are involved in the activation of PCD by ETI. In addition, the KTI protein, which may be involved in the suppression of the H_2_O_2_ accumulation, was also induced at a late time point, suggesting a fine-tuning mechanism in this pathogenesis stage to control the H_2_O_2_ mediated HR and PCD responses.

### 2.6. Changes Associated with the Regulation of Phytohormones

Several proteins involved in the biosynthetic or signaling pathways of different phytohormones, including SA, ethylene (ET), and abscisic acid (ABA), were identified in our study (Table 3). Three lipoxygenases involved in JA biosynthesis were regulated in various ways at different post-inoculation time points. Tomato lipoxygenase D (LoxD; Solyc03g122340) was downregulated with a fold change of 0.65 at 24 hpi but not significantly regulated at either 48 or 96 hpi. LoxD was one of the first proteins to be identified from the JA biosynthetic pathway, and mediates defense against fungal and bacterial pathogens [50,51]. In contrast, the other two lipoxygenase proteins (Solyc01g099160 and Solyc08g029000) were upregulated with a 3.7 to 4.19-fold increase at 48 hpi or a 13.97-fold increase at 96 hpi, respectively. In addition to the proteins involved in JA biosynthesis, topless 3 (TPL3; Solyc01g100050), a transcriptional corepressor of the JA responsive genes [52], was upregulated by 1.76-fold at 96 hpi. It has been speculated that TPL protein is involved in multiple pathways and interacts with numerous transcriptional repressors [53]. Notably, the repression of JA responses by TPL3 is dependent on its interaction with another repressor, Novel Interactor of JAZ (NINJA). TPL3 and NINJA are recruited together by the jasmonate ZIM domain (JAZ) protein to inhibit the expression of the early JA-responsive genes [52]. The transcriptional regulation by TPL and family members has been shown to be a crucial part of the immune signaling for defense against both biotrophic oomycetes and necrotrophic fungus [54]. 1-aminocyclopropane-1-carboxylate (ACC) oxidase (ACO1; Solyc07g049530), functioning as the rate-limiting enzyme of the ET biosynthesis, was upregulated by *P. infestans* at 48 and 96 hpi with 1.84 and 4.88-fold increases, respectively. CobN/magnesium chelatase (CHLH/ABAR; Solyc04g015750) was upregulated in abundance by 1.55-fold at 24 hpi, but downregulated with a fold change of 0.42 at 96 hpi. CHLH/ABAR is involved in the plastid-to-nucleus retrograde signaling for chlorophyll development, and stomatal response to ABA, interacting with a group of WRKY transcription factors such as WRKY40 thus attenuating their negative regulation on ABA-responsive genes [55,56,57].

Taken together, repression of the biosynthetic pathway of JA and enhancement of the ABA signaling pathway were observed at 24 hpi with *P. infestans*. The repression of JA production may facilitate the activation of SA-related responses to defeat the biotrophic infection of *P. infestans*. This JA repression may facilitate *P. infestans* transition from biotroph to necrotroph. The enhanced ABA signaling may benefit *P. infestans* infection at the early time point as ABA induces disease susceptibility to various biotrophic or necrotrophic pathogens in a wide range of plant species [58]. *P. infestans* also suppressed the JA responsive genes at the later time point of 96 hpi, while plants may increase the level of JA and ET from 48 hpi to 96 hpi as well as downregulate ABA signaling at 96 hpi. Since the activation of JA and ET production is involved in the enhanced plant resistance against necrotrophic pathogens [6]; these results suggest that plants try to trigger the pathogen resistance from the biotrophic to necrotrophic pathogenesis stage via adjusting the signaling and/or biosynthesis of JA, ET, and ABA.

### 2.7. Changes Associated with Reactive Oxygen Species and Oxidation–Reduction Reactions

In this study, we found that 25 proteins involved with reactive oxygen species (ROS) or redox function were differentially regulated in response to *P. infestans* pathogenesis (Table 4 and Table S1). The NADPH/respiratory burst oxidase protein D (RbohD) homolog, whitefly-induced p91-phox (GP91^phox^, Solyc03g117980) protein was upregulated at 48 and 96 hpi by 1.70 and 5.08-fold, respectively. The plant NADPH oxidase RbohD is a primary player responsible for the ROS burst after the pathogen-associated molecular pattern (PAMP) perception and thus regulates the HR in and around the infection site [59]. Twelve enzymes working as ROS scavengers were up- or downregulated in this study, including 9 proteins with peroxidase (POX) enzyme activities (Solyc08g069040, Solyc04g073990, Solyc01g006300, Solyc10g076240, Solyc04g071900, Solyc02g079500, Solyc02g092580, Solyc04g071890, and Solyc03g006700). Among them, only one POX (Solyc08g069040) had downregulation at 48 hpi with a fold change of 0.56 while the rest were upregulated at 96 hpi with a fold change from 1.61 to 13.28. The other ROS scavenger enzymes regulated at 96 hpi were catalase (CAT; Solyc04g082460) with a fold-change of 0.56, superoxide dismutase 1 (CSD1; Solyc01g067740) with a 2.16-fold increase, and glutathione peroxidase-like encoding 1 (GPX-1; Solyc08g080940) with a 1.94-fold increase. In addition, another protein in the antioxidant redoxin family, thioredoxin (TRX; Solyc04g081970), was downregulated with a fold change of 0.47 at 96 hpi. Thioredoxin reductase (TRXR; Solyc02g082250) for catalyzing the reduction of TRX was upregulated by 1.52-fold. Another protein predicted to be involved in TRX regulation, CBS domain-containing protein-like (CDCP-like; Solyc01g107860) was 2.10-fold upregulated at 96 hpi. Seven proteins participating in the regulation of the homeostasis of the antioxidant glutathione (GSH) showed different patterns of regulation across the pathogen growth stages. Among them, four glutathione S-transferases (GST) or GST-like proteins (Solyc06g009020, Solyc08g080900, Solyc10g084400, and Solyc09g011590) for conjugation of the reduced form of GST to the xenobiotic substrate were increased at 96 hpi by 1.58 to 7.42-fold while two GSTs (Solyc06g083770 and Solyc02g081430) were downregulated with a fold change of 0.41 and 0.58, respectively. On the other hand, gamma-glutamylcyclotransferase (GGCG; Solyc11g012910) which is responsible for the degradation of GSH in the cytosol [60] was downregulated with a fold change of 0.68 and 0.33 at 24 hpi and 96 hpi, respectively.

In summary, of all the proteins involved in the ROS/redox homeostasis regulation, the majority of the increase in ROS burst likely occurs from 48 to 96 hpi. *P. infestans*-inoculated tomato plants also showed upregulated antioxidants and ROS scavenger enzymes at 96 hpi. The enhancement of GST proteins may be correlated with the detoxification of the effectors produced by the pathogen and the suppression of the GSH catabolic proteins may help increase the antioxidant and GST capabilities in the cytosol. During the necrotrophic pathogenesis stage, the specifically downregulated CAT, POX, or TRX could be host targets manipulated by the pathogen to favor its own growth.

### 2.8. Differentially Regulated Proteins Involved in Carbohydrate and Energy Metabolism

Proteins that are involved in carbohydrate and energy metabolism were significantly upregulated at 96 hpi (Table 5 and Table S1). Proteins participating in the glycolysis pathways, including ATP-dependent 6-phosphofructokinase (PFK; Solyc07g045160), glyceraldehyde-3-phosphate dehydrogenase (GAPDH; Solyc10g005510), and pyruvate dehydrogenase (PDH; Solyc03g097680) were all significantly upregulated at 96 hpi by 2.07 to 2.38 fold. In contrast, enolase (ENO1; Solyc03g114500) was increased earlier, at 24 hpi, with a fold change of 1.52. This enzyme catalyzes the generation of the immediate precursor of pyruvate in the glycolytic pathway and a branch point to the shikimic acid pathway. Three proteins that are involved in the pentose phosphate pathway (PPP) for producing NAPDH, transaldolase (TAL; Solyc11g033288), ribose 5-phosphate isomerase A (Rpi; Solyc05g008370), and 6-phosphogluconate dehydrogenase (6PGD; Solyc04g005160) were upregulated at 96 hpi by 1.59 to 3.70-fold. Proteins participating in fatty acid/lipid metabolism, acyl-CoA dehydrogenase (ACAD; Solyc10g076600) and enoyl-CoA hydratases (ECH; Solyc01g059830 and Solyc12g094450), were upregulated at 96 hpi with fold changes between 1.68 and 4.24. Seven proteins that function in the TCA cycle, pyruvate dehydrogenase complex (PDC; Solyc11g007720), malic enzyme (ME; Solyc08g066360), citrate synthase (CSY; Solyc01g073740), aconitases (ACO; Solyc07g052350 and Solyc12g005860), isocitrate dehydrogenase (IDH; Solyc01g005560), oxoglutarate dehydrogenase complexes (ODC; Solyc07g064800 and Solyc12g005080), succinyl-CoA ligases (SCoAL; Solyc06g083790 and Solyc01g007910) and fumarase (FUM; Solyc09g075450), were all upregulated at 96 hpi with a fold change ranging from 1.52 to 2.00. In contrast, all the proteins involved in carbon fixation and photosynthesis progress (Table 6 and Table S1), such as chlorophyll a-b binding protein (LHCB; Solyc06g063370), cytochrome b6-f complex iron-sulfur subunit (Solyc12g005630), photosystem I (PSI) P700 chlorophyll a apoprotein (Solyc06g009940), PSI reaction center subunit III (Solyc02g069450), PSI reaction center subunit N (Solyc08g013670), PSII 22 kDa protein (Solyc06g060340) and sedoheptulose-1,7-bisphosphatase (SBPase; Solyc05g052600), were downregulated at 96 hpi with fold changes between 0.54 and 0.66.

The upregulation of PFK, GAPDH, ENO1, and PDH for glycolysis together with ACAD and ECH in the fatty acid and lipid metabolisms may enhance the synthesis of acetyl-CoA for feeding the TCA cycle. TAL, Rpi, and 6PGD that function in the PPP were enhanced, and thus were likely to produce more NADPH and ribose 5-phosphate (R5P) for the alternative progress of glycolysis. The proteins involved in the TCA cycle to generate more NADH and FADH_2_ were also significantly upregulated. The increase of NAPDH by these pathways may be related to the enhancement of the oxidative phosphorylation pathway in order to generate more energy. These results suggest that plants may attempt to generate more ATP by the glycolysis/TCA/PPP and oxidative pathways during the pathogenesis process. The carbohydrate metabolites produced from the glycolysis and fatty acid biosynthetic pathways may also be used as the signals for the activation of defense responses [61]. In contrast, proteins participating in photosynthesis and energy storage were downregulated in this process, suggesting a switch in the primary metabolisms wherein carbon is obtained from the sink tissue, rather than the source.

### 2.9. Changes Associated with Secondary Metabolites

Three of the proteins involved in the secondary metabolite biosynthesis pathway were upregulated at 96 hpi (Table 7 and Table S1). Two proteins involved in the phenylpropanoid biosynthetic pathway, cinnamate 4-hydroxylase (C4H; Solyc06g150137) and 4-coumarate:CoA ligase (4CL; Solyc03g117870) were increased by 2.62- and 4.90-fold at 96 hpi, respectively. In addition, 5-enolpyruvylshikimate-3-phosphate synthase (ESPS; Solyc01g091190), which is involved in the synthesis of phenylalanine starting the phenylpropanoid pathway, was increased by 2.24-fold at 96 hpi.

We further observed that *P. infestans* induced the proteins responsible for the synthesis of the upstream and intermediate metabolites of the phenylpropanoid pathway during the necrotrophic pathogenesis stage. These upregulated proteins may enhance the level of phenylpropanoids in plants to fight against pathogens [62]. Furthermore, lignin, suberin, or flavonoids which are derived from this pathway also contribute to the basal immunity response of the plant [63].

### 2.10. Novel P. infestans-Regulated Tomato Reponses Revealed by Time-Lapse Proteomics Studies

To date, only a limited number of studies have focused on the tomato proteome in response to *P. infestans*. A previous proteomics study discovered a total of 56 tomato proteins regulated by *P. infestans* infection, of which 39 and 17 were found in the resistant and susceptible tomato genotype, respectively [15]. In the resistant tomato genotype, six proteins associated with immune and defense mechanisms were observed with enhanced abundance after pathogen inoculation, which is comprised of three peroxiredoxins, one PR protein, carboxypeptidase, and predicted R protein. Two proteinase inhibitors and a glucosidase were observed to have suppressed abundance in the resistant tomato genotype. In the susceptible genotype, among the stress and defense-related proteins, one enzyme participating in the urea cycle and metabolism of amino groups was suppressed whereas one ROS scavenger enzyme and one glucosidase were both enhanced at the protein level after infection. Compared to the previous proteomics study, we identified 25-fold more tomato proteins regulated by this pathogen; thus most of the *P. infestans*-regulated mechanisms were first identified and dynamically quantified at the protein level. To the best of our knowledge, this study provides the most comprehensive proteome information available to date, revealing the dynamics of tomato proteome regulation at the defined key stages of *P. infestans* infection. With regard to the dynamics of tomato defense responses, we discovered that PR-5 protein was downregulated at the biotrophic stage, and multiple chitinases were induced to conduct the defense activity by degrading the pathogen cell wall in the necrotrophic stage. In addition, tomato proteins for direct antipathogen activity, biosynthesis and signaling of immune regulatory hormones, ETI responses, and biosynthesis of the secondary metabolites were also exclusively determined to be regulated by *P. infestans* infection in this study.

**Figure 4:**
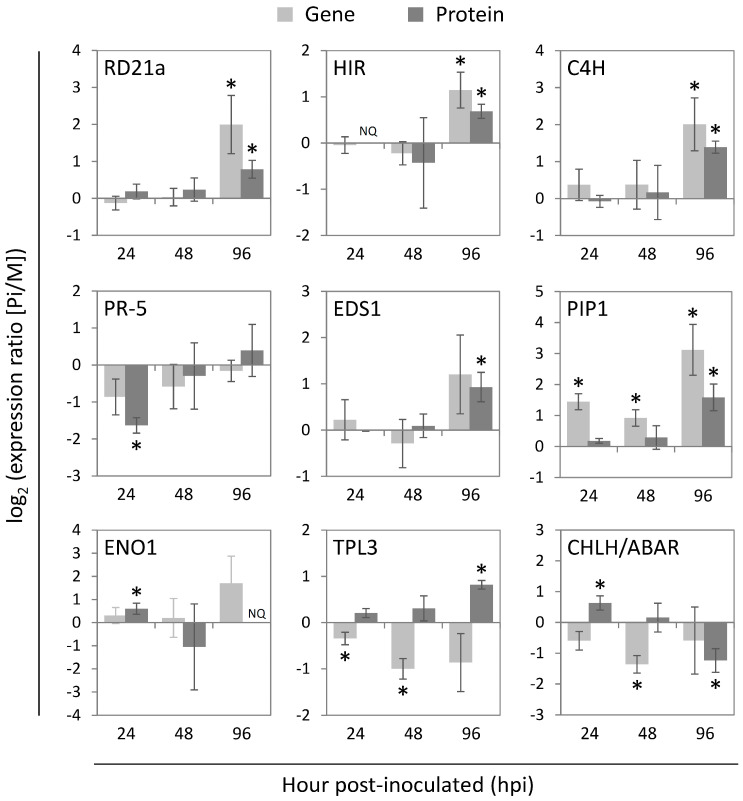
Levels of tomato transcripts encoding proteins that were differentially regulated in response to different pathogenesis stages of *P. infestans*. The transcript levels of the selected *P. infestans*-regulated proteins (RD21a, HIR, C4H, PR-5, EDS1, PIP1, ENO1, TPL3, and CHLH/ABAR) in tomato were determined by qRT-PCR in the same samples prepared for the proteomics analysis in this study. The mean values from three biological repeats are shown (n = 3). All statistically significant differences between the *P. infestans* (Pi) and mock (M)-treated samples are indicated with asterisks (*, *p* < 0.05), based on the Student’s *t*-test using the log_2_-ratio of the gene or protein expression levels between these two conditions. The internal control *Ubi-3* gene was used for normalization. Error bars are means ± SD. NQ, non-quantifiable as the protein quantification was only measurable in one or two biological replicates.

Although the response to *P. infestans* has been previously analyzed in tomato in well-defined stages of pathogenesis using a RNA-Seq-based transcriptomics approach [11], this study lacks a mock-treated sample as a control at each time point; this may lead to identifying non-pathogenetic responses. In addition, the regulation at the protein level also requires investigation to further clarify the roles of these gene candidates. With the mock-treated sample as the control at each time point, many of the *P. infestans*-responsive genes identified in the previous RNA-Seq analysis were statistically proved to be regulated at the protein level in this study. However, it is well-known that gene expression is not always proportional to protein expression, and accordingly we discovered there were some proteins differentially regulated by *P. infestans* infection that were reported differently in the previous transcriptomic study. Among them, nine proteins identified in this study were either not identified (C4H), not significantly expressed (TPL3), or showed opposite regulation patterns at the transcript level (PR-5, RD21a, EDS1, HIR, PIP1, CHLH/ABAR, and ENO1). These proteins have molecular functions in direct antifungal activity (PR-5), cysteine protease (RD21a and PIP1), R protein helper inducing HR (HIR), SA/ETI-mediator required for basal and NB-LRR protein-induced resistance (EDS1), JA/ET signaling (TPL3), ABA signaling (CHLH/ABAR), glycolysis (ENO1), and the phenylpropanoid biosynthetic pathway (C4H). For these selected proteins, their gene expressions were analyzed by qRT-PCR in the same biological samples used in this proteomics study (Figure 4).

Among those nine proteins, three proteins, RD21a, HIR, and C4H showed positively correlated regulations between the transcript and protein levels. Four proteins, PR-5, EDS1, PIP1, and ENO1 showed similar trends of direction in gene and protein regulation. In the case of PIP1, differential expression at the protein level was observed at a later time point (96 hpi) than the transcript level (24 and 48 hpi). There could be more variation in the expression of these four genes; thus the changes of transcript levels during the pathogen infection did not reject the null hypothesis in the significant test. The higher variation in transcript regulation than protein regulation might also explain the non-correlation of results in our proteome analysis and the previous RNA-Seq analysis. As the previous transcriptomic analysis only used one biological replicate, the actual changes in the transcript levels after pathogen infection may not be accurately presented. The non-correlation of the results of the transcriptome and proteome analysis could be due to the biosynthesis activity and stability of RNA and protein under differential regulation [64]. We found that two proteins TPL3 and CHLH/ABAR showed opposite directions of transcriptional and translational/post-translational regulation after pathogen infection. This opposite regulation trend could result from multiple factors beyond the transcript concentration, which influence the establishment of the protein expression level. These factors could include the modulation of translation rate by mechanisms like regulator elements/miRNA and constraints of machinery/resources for protein biosynthesis, the modulation of protein half-life by the ubiquitin-proteasome or autophagy pathway, the temporal delay in protein synthesis, and the protein export resulting in spatial changes of proteins [65].

When examining how these proteins and their gene levels were regulated in the three pathogenesis phases, we found that although PR-5 protein was downregulated at 24 hpi, its gene expression level was not significantly changed. In addition, at this early stage, although ENO1 and CHLH/ABAR protein levels were increased, their coding genes appear to be non-differentially expressed. On the other hand, the protein levels of neither PIP1 nor TPL3 were regulated although the transcript levels of *PIP1* and *TPL3* were enhanced and suppressed at 24 hpi, respectively. Later at 48 hpi, the transcript levels of *PIP1* gene showed upregulation, *TPL3* and *CHLH/ABAR* genes showed downregulation whereas none of their protein levels was significantly altered from the mock-treated group. At 96 hpi, EDS1 and TPL3 were both upregulated, and CHLH/ABAR protein was downregulated but neither of their gene expressions was differentially regulated. On the other hand, RD21a, HIR, C4H, and PIPI1 were regulated at both the transcript and protein levels at 96 hpi.

Since TPL3 functions as the transcription corepressor of JA-responsive genes, the reduction of TPL3 protein level may lead to the activation of JA signaling, which may suppress the production of SA-mediated immune responses. In this study, the suppression of *TPL3* gene expression may be part of the infection strategy of *P. infestans*, which deactivates the SA-mediated immunity to establish a biotrophic interaction with the host plant at the early infection stage. However, the suppressed *TPL3* transcript did not result in suppression of the protein level, possibly due to the translational activity or protein stability being enhanced to compensate for the repression of *TPL3* gene. This may facilitate plants to activate the SA signaling to fight against the biotrophic pathogen. In addition, PR-5 protein, which could directly sabotage the pathogen cell wall assembly, may be suppressed by *P. infestans* specifically at the protein level but not at the transcript level. Also, the increased *PIP1* gene expression did not result in significant protein abundance change in the biotrophic phase. The PIP protease activity has been reported to be a target of *P. infestans* effectors; therefore, this inhibition may cause PIP1 protein to become more unstable, thus generating a lower induction fold at the level of PIP1 protein in comparison with its transcript. Another possible defense strategy may include CHLH/ABAR protein for its role of controlling stomatal closure [66] and relieving the expression of ABA-responsive genes [57], hence the increased CHLH/ABAR protein in the biotrophic phase may represent an early PTI response. On the other hand, since ENO1 is a glycolytic enzyme, the upregulated ENO1 protein could be interpreted as one of the plant early responses to gain more energy or as a pathogen strategy to obtain more carbohydrate nutrients during the biotrophic phase. This translational/post-translational but not transcriptional regulation of ENO1 indicates an urgent response to facilitate the glycolysis pathway.

In the transition phase, the suppressed *TPL3* transcript and enhanced *PIP1* transcript without significant changes in protein levels suggest the continuous action of the pathogen in repressing these immune regulators from the early biotrophic phase. In the meantime, the plant may still try to enhance its immune responses by activating the transcriptional or translational regulation of these genes. In addition to controlling stomatal closure, CHLH/ABAR also influences ABA sensitivity [66,67,68,69,70] and contributes to chloroplast development [71]. Therefore, the suppressed expression of the *CHLH/ABAR* gene without the protein abundance being regulated in the transition phase suggests *P. infestans* may have started to hijack the ABA signaling pathway and disrupt plant growth to prepare itself to initiate necrosis, as well as inhibit stomatal closure, for the next pathogenesis stage.

Although the gene expression of *TPL3* was repressed from the early to the late pathogenesis stages, TPL3 protein was significantly upregulated in the necrotrophic phase. This regulation of TPL3 protein level was able to suppress the JA-responsive genes, which is favorable for *P. infestans* to facilitate the necrotrophic lifestyle. The downregulated CHLH/ABAR protein level but not the transcript level in the necrotrophic phase may reflect a delay effect from the transcriptional regulation in the transition phase. In view of the function of CHLH/ABAR as mentioned above, in the necrotrophic phase, this filamentous pathogen may downregulate CHLH/ABAR protein level to inhibit the stomatal closure to help the new sporangiophore emerge from the stomata and produce sporangia which are spread by wind or water to infect new plants [72]. As PIP1 and RD21a are both proteases for triggering PCD, their upregulated transcription and protein levels in the necrotrophic phase may have different causes: (1) a delay in protein translation production, as there is a time delay from transcriptional induction to protein level increase following an induced state change; (2) the plant eliminating the suppression from pathogen effectors which target these defense proteins starting in the early infection stage; (3) the pathogen acting to induce necrosis by secreting various cell-death inducing effector proteins to thrive on dead host tissues [73]. As both EDS1 and HIR contribute to the R protein (especially NB-LRR)-induced immunity, the upregulated EDS1 and HIR proteins in response to the necrotrophic phase of *P. infestans* suggests a plant action to increase the local concentration of NB-LRR proteins, and possibly other R proteins to enhance the detection sensitivity of pathogen effectors.

## 3. Materials and Methods

### 3.1. Plant Material, Growth Conditions and P. infestans Inoculation

Tomato seeds (*Solanum lycopersicum* cv CL5915, originally provided by AVRDC—The World Vegetable Center, Tainan, Taiwan) were planted in soil and grown in a growth chamber for 5 weeks under a 12 h/12 h light-dark cycle, with 50%/70% (light/dark) humidity, 25 °C/20 °C (light/dark) temperature and a light source providing photosynthetic photon flux density (PPFD) of 100 µmole/m^2^∙s. Fully expanded leaflets with similar size from the 3rd or 4th pair of true leaves were collected for the following experiment. Three leaflets from three individual plants were pooled as one biological replicate at each time point for the pathogenicity assays. *P. infestans* strain was sub-cultured every two weeks and grown on fresh Rye A agar plates [74] in the forever dark incubator at 20 °C for 2–3 weeks before collecting sporangia. By using a cell spreader, sporangia were washed in sterilized distilled water, and after counting under the optical microscope at a magnification of 200X, the concentration was adjusted to 2.0 × 10^4^ sporangia per ml for inoculations. One leaflet from each pair was separately placed in the mock- or pathogen-inoculated group and placed on the distilled water-saturated tissue paper in square Petri dishes before inoculation. The abaxial surface of a tomato leaf was inoculated with 8 droplets of 20 µL sporangial suspension. The mock-treated groups had the same treatment except that the sporangial suspension was replaced with distilled water. After treatment, the dishes containing the leaflets were sealed with Parafilm and incubated in the dark at 20 °C. Samples were collected at 6, 16, and 24 hpi for microscope observation, 24, 48, and 96 hpi for RNA and protein extraction and 24, 48, 96, and 120 hpi for disease phenotype observation.

### 3.2. Observation of P. infestans Growth by Trypan Blue Staining and Microscope

To visualize the *P. infestans* infection at the early time points, leaves at 6, 16 or 24 hpi were transferred to a beaker and immersed in trypan blue solution (10 g phenol, 10 mL glycerol, 10 mL lactic acid, 10 mL water and 10 mg of trypan blue) diluted with 2-fold of ethanol [75]. The beaker was then heated in boiled water until the staining solution boiled for about 2 min and the leaves turned color to light blue. After being placed at room temperature for about 8 h for complete dyeing, the leaves were destained by replacing the staining buffer with chloral hydrate solution (5 g of chloral hydrate dissolved into 2 mL of distilled water) and shaken at a slow speed of 30 rpm for 24 h [76]. Lastly, the leaf samples were observed under a Zeiss AxioImager Z1 high-performance research microscope (Carl Zeiss AG, Werk Göttingen, Germany) with differential interference contrast optics.

### 3.3. RNA Extraction and Marker Gene Expression Analysis of P. infestans-Inoculated Tomato Leaf

Total RNA was extracted from each sample by using the Total RNA Mini Kit Plant (Geneaid, New Taipei City, Taiwan), according to the manufacturer’s protocol. RNA was quantified by using Nanodrop ND-1000 (NanoDrop Technologies/Thermo Scientific, Wilmington, DE, USA), and 4 µg of total RNA was employed for cDNA synthesis in a 20-μL volume by using SuperScript™ III Reverse Transcriptase (Thermo Scientific, Bellefonte, PA, USA), following to the manufacturer’s protocol. PCR was carried out with 20 ng of the cDNA in a 20-μL volume containing 2 μM of each specific primer (Supplemental Table S3) and 2X SuperRed PCR Master Mix (Biotools, New Taipei City, Taiwan), following the manufacturer’s protocol.

For RT-PCR analysis, the PCR condition consisted of one cycle of 95 °C for 5 min, followed by 35 cycles of a three-step loop (94 °C for 1 min, 55 °C for 1 min and 72 °C for 1 min) and a final step of 72 °C for 5 min. After gel electrophoresis, the relative intensities of the product bands were assessed using ImageJ software [77]. The relative intensities of gel bands were calculated by the band with the maximal intensity as the value of 1.

The qRT-PCR was used to validate the expression of specific tomato genes. Three biological replicates were used for qRT-PCR analysis, which was performed using SYBR Green reagent (Sigma-Aldrich) and ABI 7500 Fast Real Time PCR system (ThermoFisher Scientific, Inc.). The PCR cycling steps were 50 °C for 2 min and 94 °C for 10 min for the initial steps followed by 95 °C for 15 s and 60 °C for 1 min for 40 cycles. The gene expressions across different samples were normalized with the internal control Ubi3. The primers used are listed in Supplemental Table S4. The melting curve was used to verify the specificity of the PCR product.

### 3.4. Sample Preparation for Proteome Analysis

Three biological replicates of mock-treated (24, 48, 96 hpt) and *P. infestans* inoculated (24, 48, 96 hpi) leaves were prepared for the proteomics experiments. For each sample, leaves were ground into powder in a chilled pestle and mortar with liquid N_2_ then 0.2 g of the powder was collected. The protein extraction protocol was modified from previous studies [78,79,80]. The sample was then homogenized with 0.9 mL of ice-cold pH 8.0 homogenization buffer (0.5 M Tris-HCl, pH 7.5; 0.7 M sucrose; 0.1 M KCl; 50 mM EDTA; 1% polyvinylpolypyrrolidone (PVPP); 5 mM dithiothreitol, 1 mM phenylmethylsulfonyl fluoride and 1× Roche protease inhibitor cocktail) by vortexing for at least 3 min or until completely homogenized. The sample was further incubated in the buffer by Intelli Mixer RM-2L (ELMI Ltd., Riga, Latvia) in the cold room for 30 min. The homogenate was filtered through two layers of Mira cloth. After collecting the supernatant of the filtrate by centrifugation at 16,000× *g* for 10 min under 4 °C, an equal volume of phenol (pH 7.8–8.0, Tris-buffered) was added to the supernatant and homogenized by Intelli Mixer RM-2L for 30 min at 4 °C. The upper phenol phase was collected after the solution was centrifuged at 16,000× *g* for 20 min then the back-extraction was performed one more time using an equal volume of phenol. Proteins were precipitated from the final collected phenol phase by mixing with a 5-fold volume of cold 0.1 M ammonium acetate/methanol and incubating at −20 °C overnight. The precipitated proteins were washed once with 0.1 M ammonium acetate/methanol and twice with 80% acetone/10 mM DTT. The air-dried protein pellet was then solubilized in 8 M urea with 50 mM ammonium bicarbonate (ABC) and 5 mM tris (2-carboxyethyl) phosphine hydrochloride (TCEP). The extracted total protein concentration of each sample was measured by the Bradford assay and checked by SDS-PAGE.

For each sample, 100 μg of the solubilized tomato total protein was reduced, alkylated and proteolyzed with lysyl endopeptidase and trypsin following the steps previously described [81]. The digested sample was then desalted using the 100-mg tC18 SepPak cartridge (Waters Corporation, Milford, MA, USA). After being quantified using the Peirce BCA assay kit (Thermo Fisher Scientific, Waltham, MA, USA), the tryptic peptides from the individual sample were dissolved by deionized water containing 2% acetonitrile and 0.1% (*v*/*v*) formic acid to a concentration of 500 ng/μL. The pooled peptide sample of the mock and *P. infestans*-inoculated samples at each time point (as QC sample) was analyzed by LC-MS/MS with the DDA mode in order to construct the DIA spectral library. For the purpose of retention time calibration, the iRT-standard peptides (Biognosys, Schlieren, Switzerland) were mixed with the pooled sample or each individual sample with a ratio of 1:10 by volume.

### 3.5. Liquid Chromatography-Mass Spectrometry Analysis

The nanoLC−MS/MS was equipped with a self-packed tunnel-frit [82] analytical column (ID 75 μm × 50 cm length) packed with ReproSil-Pur 120A C18-AQ 1.9 μm (Dr. Maisch GmbH, Germany) at 40 °C on a nanoACQUITY UPLC System (Waters Corporation, Milford, MA, USA) connected to a Q Exactive HF Hybrid Quadrupole-Orbitrap mass spectrometer (Thermo Scientific, Bellefonte, PA, USA). The peptides were separated by a 135-min gradient using the mobile phases, including Solvent A (0.1% (*v*/*v*) formic acid) and Solvent B (acetonitrile with 0.1% formic acid). With a flow rate of 250 nl/min, the gradient started with a 40 min equilibration maintained at 2% of B and set as the following segments: 2% to 8% of B in 8 min, 8% to 25% of B in 90 min, then 25% to 48% of B in 5 min, 48% to 80% of B in another 5 min followed by 80% of B wash 10 min and the last equilibrium to 2% B in the last 15 min.

The instrumentation and parameters for DDA and DIA analysis followed previous studies using the Q Exactive HF Hybrid Quadrupole-Orbitrap mass spectrometer [83,84]. Two micrograms of the pooled and individual tryptic peptide samples were analyzed by DDA and DIA mode, respectively. For DDA analysis, the MS instrument was operated in the positive ion mode and two different DDA methods at the MS2 level. In the first DDA method, full-MS was acquired with high resolution (R = 60,000 at *m*/*z* 200 at an automatic gain control target of 3.0 × 10^6^) and broadband mass spectra (m/z 350–1650 Da) with a maximum IT of 15 ms, and MS/MS events (R = 15,000 at an automatic gain control target of 1.0 × 10^5^) with a dd-MS² IT of 45 ms when a precursor ion charge was 2+, 3+, 4+ and 5+ and minimum AGC target as 4.5 × 10^3^, isolation window was set to 1.2 Th, was detected. The 20 most abundant peptide molecular ions, dynamically determined from the MS1 scan, were selected for MS/MS using stepped normalized collision energies (NCE) as 26.5%, 28%, and 29.5%, with the MS/MS settings of dynamic exclusion as 25 s, peptide matched as preferred, isotopes excluded and apex triggered. The other DDA method was using gas-phase separation with the m/z range of 350–510, 500–710, 700–1060 and 1040-1650 Th and fixed NCE as 28%; the rest of settings were the same as the first DDA method.

For DIA analysis, MS/MS proteome profiling was analyzed by the same LC−MS/MS system. The instrument was operated in the positive ion mode and configured to collect high resolution (R = 120,000 at m/z 200 at an automatic gain control target of 3.0 × 10^6^) broadband mass spectra (*m*/*z* 350–1650 Da) with a maximum IT of 60 ms, and MS/MS events (R = 30,000 at an automatic gain control target of 3.0 × 10^6^) with an auto MS² IT, isolation window was set to 52.0 *m*/*z*, fixed first mass was set to 200 *m*/*z*. The 25 segments were selected for MS/MS using a higher energy collisional dissociation (HCD) energy of 28%. The acquisition window covered a mass range from 350 to 1650 *m*/*z* through 25 consecutive isolation windows. In each set of DDA or DIA data, the iRT calibration was required with a minimum R^2^ of 0.9 by a manual check. During the DIA LC-MS/MS runs across the sequence of the instrument in the study, the coefficient of variation (CV) of iRT peptide retention time was monitored to be less than 3% and the CV of iRT peptide peak intensity to be less than 20% in the QC samples run in-between the DIA analysis of each sample.

### 3.6. MS Data Analysis

With all the DDA data, Mascot (ver. 2.3, http://www.matrixscience.com/, accessed on 10 April 2021) and X!Tandem (ver. 2013.06.15.1) [85] were used to do a protein database search against a combined database of *Solanum lycopersicum* proteome (ver. 4.1 accessed 16 July 2020; downloaded from the website ftp://ftp.solgenomics.net/tomato_genome/annotation/ITAG4.1_release; 34688 entries; the reverse sequences generated as the decoy database), *P. infestans* proteome (released in April 2020; accessed 17 August 2020; downloaded from the NCBI website ftp://ftp.ncbi.nlm.nih.gov/genomes/genbank/protozoa/Phytophthora_infestans/latest_assembly_versions/GCA_012295175.1_ASM1229517v1, accessed on 10 April 2021; 20172 entries; the reverse sequences generated as the decoy database), sequences of PR-1b protein (Solyc00g174340) which is missing from the ITAG 4.1 database, the iRT standard peptides and BSA (SwissProt Accession: P02769). Search parameters were set as follows: MS tolerance, 10 ppm; precursor monoisotopic mass isotope (M + 1), included; the number of trypsin missed cleavage, 2; fragment mass tolerance, 0.1 Da; enzyme, trypsin/P; fixed modifications, carbamidomethyl cysteine (+57.021 Da); variable modifications, oxidized methionine (+15.995 Da) and acetyl protein N-terminus (+42.016 Da). Next, the search identifications from different search engines and different repeats were combined and statistically scored using PeptideProphet [86] and iProphet [87] within the Trans-Proteomic Pipeline (TPP, ver. 5.2) [88]. MAYU [89] was used to select an iProphet cutoff of 0.913764, resulting in a protein FDR of 1%. SpectraST [90] was used in library generation mode with HCD settings. In the constructed spectral library, there were a total of 65,763 transitions, 53,085 peptides and 11,563 proteins. OpenMS (ver. 2.5) [91] was utilized for decoyed spectral library construction with the workflow adapted from the previously published research [92]. To be included in the final spectral library, product ions were required to contain a minimum of seven amino acids in peptide length and only the best six product ions were selected for each peptide.

The DIA data were analyzed using OpenSWATH (ver. 2.4.0) [93] software against the constructed spectral libraries to identify and quantify peptides and proteins. The retention time alignment used the information of iRT transitions (RT tolerance was set as 7 min for DIA data). In addition to the chromatogram alignment, the spike-in iRT peptide standards were also used for the quality control of the DDA and DIA analyses. Parameters used for feature alignment were set as follows: peptide false-discovery rate (FDR), 0.05; protein FDR, 0.01; alignment method, Local MST; re-alignment method, lowest; retention time (RT) difference, 30 s; alignment score, 0.05. The ratios of protein quantitation between the *P. infestans*-inoculated and mock-treated samples in each replicate were normalized by the most-likely ration normalization principle as previously applied in a DIA study [94]. The mass spectrometry proteomics data have been deposited to the ProteomeXchange Consortium via the PRIDE [95] partner repository with the dataset identifier PXD022266.

### 3.7. Quantitation Data Analysis

Peptide quantity was exported from the analysis result from OpenSWATH using the measurement by the sum of six best precursors and the peptide ratio was calculated by the peptide quantity of the *P. infestans*-inoculated and mock sample. The protein quantity ratio was calculated by the weighted geometric mean of the unique and/or shared peptide ratios using the peptide quantity as the weighting factor. The criteria for selecting unique and shared peptides for protein or protein group quantification were as follows: (1) if a protein is identified with unique and shared peptides, only the unique peptides will be used; (2) if more than 2 proteins are grouped based on shared identified peptides, both unique and shared peptides will be used as this protein group quantity and any protein without unique peptide identified in this group will be listed in the subset; and (3) if more than 2 proteins are grouped but no unique peptide is identified, then all the shared peptides will be used as this protein group quantity. As the mock and pathogen inoculated detached leaves were collected from paired leaflets of the same plant and samples of three individual plants were pooled in each condition, the initial abundances of proteins (if without treatment) for all conditions at the same growth stage were considered as identical. Thus, a paired Student’s *t*-test (one sample, null hypothesis, no change, mean μ = 0) was performed to uncover differential expression between control and *P. infestans*-inoculated sample for the same growth stage of plants. The *t*-test was performed based on the protein log_2_ ratios of the *P. infestans*- and mock-inoculated sample from three biological replicates. Proteins with a log_2_ fold-change of higher than 0.58 or lower than -0.58 and a *p*-value of less than 0.05 were considered as significant up- or downregulated proteins. The candidate proteins were searched against the *Arabidopsis thaliana* genome assembly *TAIR10* from The Arabidopsis Information Resource (TAIR) database (http://arabidopsis.org) using Protein Basic Local Alignment Search Tool (BLASTP) with E-value < 1.0 × 10^−5^ (https://blast.ncbi.nlm.nih.gov/Blast.cgi; ver. 2.7.1, accessed on 18 December 2018) first, and these Arabidopsis homologs were then submitted to the web-based platform of the Database for Annotation, Visualization and Integrated Discovery (DAVID; http://david.abcc.ncifcrf.gov, v6.8, accessed on 10 April 2021) for Gene Ontology (GO) enrichment and function analysis.

## 4. Conclusions

In this study, we investigated the dynamics of the tomato proteome regulation under *P. infestans* biotrophic, biotrophic-necrotrophic transition, and necrotrophic infection stages using a DIA-based quantitative proteomics approach. By constructing a tomato peptide spectral library containing over 11,000 tomato proteins using 2D-LC-MS/MS, a total of 6631 tomato proteins were identified by DIA analysis. At the stage of biotrophic infection, tomato proteins involved in glycolysis, fatty acid/lipid biosynthesis, and ABA signaling were shown to be upregulated whereas proteins involved in direct defense, redox homeostasis, and JA signaling were repressed. The regulated mechanisms may help the pathogen to establish a compatible interaction with the host and obtain more nutrients from the plant cells. From the biotrophic to transition pathogenesis, proteins involved in JA/ET biosynthesis, JA/SA immune signaling, regulation of HR, and defense function were upregulated, whereas proteins involved in ROS production were suppressed. This implies that the immune responses were activated at this stage, but the ROS production may be controlled. When proceeding to the stage of necrotrophic infection, the plant proteome was dramatically affected with regard to most of the resistance-related proteins, including the ones with direct defense functions, immune regulators mediating HR and PCD, and the biosynthetic enzymes of defensive secondary metabolites, which were all upregulated. At this stage, photosynthesis and protein synthesis machinery were ubiquitously downregulated, whereas energy-generation metabolisms were increased, suggesting plants may transfer to the production of energy and regulation as a trade-off between the growth and defense response. Throughout these different pathogenesis stages, the regulation of the defensive phytohormones, including SA, JA, ET, and ABA, possibly caused a synergistic or antagonist effect, and the regulation of the ROS/redox homeostasis appeared to be dynamic during the pathogenesis progression. Several proteins with differentially regulated protein levels across different pathogenesis phases were selected to examine their gene expression levels. Among them, we found that several candidates like PR-5, ENO1, TPL3, PIP1, and CHLH/ABAR could be key targets of *P. infestans* to suppress the host defense/immunity via the transcriptional or translational/post-translational regulation during different infection stage.

It is worth noting that in the early infection stage, it may be essential for *P. infestans* to regulate the protein levels of TPL3, PR-5, and PIP1 to establish a compatible interaction. While in the later infection stages, the regulation of translation and protein stability of TPL3 may be crucial for the pathogen to establish its necrotrophic lifestyle. Hence, this study provides comprehensive information about the regulation in tomato proteome under different pathogenesis stages of *P. infestans*. The translational or post-translational regulation mechanisms of these proteins may also inform the development of a better strategy to control late blight disease in this crop.

## Figures and Tables

**Table 1:** Proteins involved in defense mechanisms with a significant change in abundance at 24, 48 and 96 h post-inoculation with *P. infestans.*

			24 h			48 h			96 h		
Gene Accession	Protein Description	Arabidopsis Homolog	# rep ^a^	log_2_ ratio ^b^	*p* ^c^	# rep ^a^	log_2_ ratio ^b^	*p* ^c^	# rep ^a^	log_2_ ratio ^b^	*p* ^c^
Solyc10g055800	chitinase	AT3G12500	3	−0.121	0.804	3	0.454	0.347	3	1.816	0.023
Solyc02g082920	class II chitinase	AT3G12500	3	0.140	0.326	3	0.556	0.009	3	2.982	0.019
Solyc01g008620	glucan endo-1,3-beta-glucosidase	AT4G16260	3	0.072	0.133	3	−0.022	0.933	3	2.563	0.001
Solyc10g079860	LEQB L.esculentum TomQ’b beta (1,3) glucanase	AT3G57270	3	0.089	0.496	3	0.612	0.344	3	4.055	0.007
Solyc09g090990	major allergen Pru ar.1 (PR-10 family)	AT5G45860	3	0.755	0.162	3	2.446	0.002	3	4.479	0.001
Solyc07g005370	norcoclaurine synthase (NCS; PR-10/Bet v 1 family)	AT2G26040	3	0.135	0.642	3	0.375	0.153	3	−0.756	0.033
Solyc08g080670	pathogenesis-related 5-like protein (PR-5)	AT4G11650	3	−1.633	0.005	3	−0.298	0.623	3	0.394	0.436
Solyc01g097240	pathogenesis-related protein 4 (PR-4)	AT3G04720	3	0.199	0.027	3	0.522	0.058	3	3.745	0.007
Solyc09g090980	pathogenesis-related protein STH-2-like (PR-10 family)	AT1G24020	3	0.185	0.009	3	1.206	0.010	3	3.456	0.004

^a^ Number of biological replicates in which the protein was quantifiable. ^b^ The average log_2_ ratio of protein quantity representing (inoculated/mock) from three biological replicates. ^c^ The *p*-value was calculated by Student’s *t*-test if the protein could be quantified from three biological replicates. Color codes: red, significant quantity change with greater than 0.58 of log_2_ ratio (*p* < 0.05); blue, significant quantity change less than −0.58 of log_2_ ratio; grey, no significant change between the inoculated and mock group (*p* ≥ 0.05) or not quantified in all three replicates (NQ); white, the quantity change is between 0.58 and −0.58 of log_2_ ratio (*p* < 0.05).

**Table 2:** Proteins involved in immune regulation with a significant change in abundance at 24, 48 and 96 h post-inoculation with *P. infestans*.

			24 h			48 h			96 h		
Gene Accession	Protein Description	Arabidopsis Homolog	# rep ^a^	log_2_ ratio ^b^	*p* ^c^	# rep ^a^	log_2_ ratio ^b^	*p* ^c^	# rep ^a^	log_2_ ratio ^b^	*p* ^c^
Solyc02g077880	auxin-repressed protein (ARP)	AT2G33830	3	−0.404	0.202	3	−1.360	0.072	3	−2.441	0.007
Solyc03g033790	P-loop containing nucleoside triphosphate hydrolases superfamily protein	AT3G50930	1	0.083	NQ	2	0.304	NQ	3	1.340	0.021
Solyc06g068840	calcium-dependent phospholipid-binding copine family protein	AT5G61900	2	0.262	NQ	3	0.534	0.181	3	1.015	0.030
Solyc02g067750	carbonic anhydrase (CA)	AT3G01500	3	1.884	0.719	3	−1.904	0.374	3	8.330	0.012
Solyc04g078540	cysteine proteinase RD21a	AT1G47128	3	0.331	0.102	3	−0.029	0.888	3	0.716	0.036
Solyc06g071280	enhanced disease susceptibility 1 (EDS1)	AT3G48090	3	−0.015	0.100	3	0.092	0.594	3	0.930	0.037
Solyc06g071050	hypersensitive-induced response protein (HIR)	AT5G62740	2	−0.334	NQ	3	−0.430	0.525	3	0.686	0.016
Solyc03g098730	Kunitz-type protease inhibitor (KTI)	AT1G17860	3	−0.607	0.220	3	0.076	0.744	3	2.740	0.008
Solyc07g043320	myosin heavy chain, embryonic smooth protein	AT2G32240	3	0.129	0.276	3	0.578	0.188	3	1.050	0.030
Solyc00g174340	pathogenesis-related protein 1b (PR-1b)	AT4G33720	3	0.058	0.484	3	0.591	0.025	3	2.902	0.008
Solyc09g007010	pathogenesis-related protein 1 (PR-1)	AT4G33720	3	0.097	0.354	3	0.411	0.302	3	2.296	0.043
Solyc02g077040	phytophthora-inhibited protease 1 (PIP1)	AT3G49340	3	0.183	0.052	3	0.291	0.316	3	1.585	0.024

^a^ Number of biological replicates in which the protein was quantifiable. ^b^ The average log_2_ ratio of protein quantity representing (inoculated/mock) from three biological replicates. ^c^ The *p*-value was calculated by Student’s *t*-test if the protein could be quantified from the three biological replicates. Color codes: red, significant quantity change with greater than 0.58 of log_2_ ratio (*p* < 0.05); blue, significant quantity change less than −0.58 of log_2_ ratio; grey, no significant change between the inoculated and mock group (*p* ≥ 0.05) or not quantified in all three replicates (NQ); white, the quantity change is between 0.58 and −0.58 of log_2_ ratio (*p* < 0.05).

**Table 3:** Proteins involved in hormone biosynthesis and signaling with significant changes in abundance at 24, 48 and 96 h post-inoculation with *P. infestans*.

			24 h			48 h			96 h		
Gene Accession	Protein Description	Arabidopsis Homolog	# rep ^a^	log_2_ ratio ^b^	*p* ^c^	# rep ^a^	log_2_ ratio ^b^	*p* ^c^	# rep ^a^	log_2_ ratio ^b^	*p* ^c^
Solyc07g049530	1-aminocyclopropane-1-carboxylate oxidase 1 (ACO1)	AT1G05010	3	0.109	0.521	3	0.877	0.007	3	2.288	0.012
Solyc04g015750	cobN/magnesium chelatase (CHLH/ABAR)	AT5G13630	3	0.629	0.042	3	0.155	0.625	3	−1.239	0.031
Solyc01g099160	lipoxygenase (Lox)	AT1G55020	0	ND	ND	3	2.067	0.003	3	3.063	0.055
Solyc08g029000	lipoxygenase (Lox)	AT1G55020	3	−0.291	0.527	3	1.889	0.003	3	3.804	0.008
Solyc03g122340	lipoxygenase D (LoxD)	AT1G17420	3	−0.611	0.046	3	−0.453	0.394	1	−1.177	NQ
Solyc12g062290	protease do-like 9 (DEGP9)	AT5G40200	3	0.133	0.268	3	−0.588	0.011	2	0.000	NQ
Solyc01g100050	topless 3 (TPL3)	AT5G27030	3	0.205	0.067	3	0.307	0.188	3	0.817	0.004

^a^ Number of biological replicates in which the protein was quantifiable. ^b^ The average log_2_ ratio of protein quantity representing (inoculated/mock) from three biological replicates. ^c^ The *p*-value was calculated by Student’s *t*-test if the protein could be quantified from three biological replicates. Color codes: red, significant quantity change with greater than 0.58 of log_2_ ratio (*p* < 0.05); blue, significant quantity change less than −0.58 of log_2_ ratio; grey, no significant change between the inoculated and mock group (*p* ≥ 0.05) or not quantified in all three replicates (NQ); white, the quantity change is between 0.58 and −0.58 of log_2_ ratio (*p* < 0.05).

**Table 4:** Proteins involved in ROS homeostasis and redox regulation with a significant change in abundance at 24, 48 and 96 h post-inoculation with *P. infestans*.

			24 h			48 h			96 h		
Gene Accession	Protein Description	Arabidopsis Homolog	# rep ^a^	log_2_ ratio ^b^	*p* ^c^	# rep ^a^	log_2_ ratio ^b^	*p* ^c^	# rep ^a^	log_2_ ratio ^b^	*p* ^c^
Solyc04g073990	annexin p34 (ANXP34)	AT1G35720	3	−0.122	0.603	3	0.107	0.883	3	0.690	0.002
Solyc04g082460	catalase (CAT)	AT4G35090	3	0.223	0.122	3	−0.182	0.724	3	−0.845	0.046
Solyc01g107860	CBS domain-containing protein-like (CDCP-like)	AT4G36910	3	−0.026	0.345	3	−0.046	0.777	3	1.068	0.010
Solyc11g012910	gamma-glutamylcyclotransferase (GGCG)	AT1G44790	3	−0.564	0.046	3	0.369	0.418	3	−1.588	0.003
Solyc08g080940	glutathione peroxidase -like encoding 1 (GPX-1)	AT4G11600	3	−0.037	0.498	3	0.241	0.319	3	0.957	0.019
Solyc06g083770	glutathione S-transferase (GST)	AT5G44000	3	−0.008	0.967	3	−0.260	0.366	3	−1.300	0.016
Solyc10g084400	glutathione S-transferase (GST)	AT5G02790	3	−0.099	0.596	3	0.221	0.100	3	1.572	0.000
Solyc09g011590	glutathione S-transferase-like protein (GST-like)	AT3G09270	3	−1.121	0.316	3	0.987	0.272	3	2.891	0.007
Solyc02g081430	microsomal Glutathione s-Transferase (MGST)	AT1G65820	3	0.082	0.263	3	0.201	0.644	3	−0.790	0.006
Solyc01g096430	NADPH:quinone oxidoreductase-like (NQR-like)	AT3G27890	2	−0.040	NQ	3	0.140	0.814	3	1.794	0.034
Solyc03g111720	peptide methionine sulfoxide reductase (MSR)	AT5G61640	3	−0.440	0.079	3	0.135	0.396	3	0.829	0.010
Solyc10g076240	peroxidase (POX)	AT5G05340	0	NQ	NQ	0	NQ	NQ	3	3.731	0.037
Solyc08g069040	peroxidase (POX)	AT4G37530	2	−0.165	NQ	3	−0.831	0.041	2	−0.428	NQ
Solyc01g067740	superoxide dismutase 1 (CSD1)	AT1G08830	3	0.320	0.060	3	0.074	0.820	3	1.113	0.038
Solyc04g081970	thioredoxin (TRX)	AT1G76080	3	0.021	0.805	3	0.000	1.000	3	−1.087	0.011
Solyc02g082250	thioredoxin reductase (TRXR)	AT2G17420	3	0.015	0.883	3	0.217	0.262	3	0.607	0.013
Solyc03g117980	whitefly-induced gp91-phox (GP91phox)	AT5G47910	3	0.039	0.777	3	0.765	0.038	3	2.345	0.016

^a^ Number of biological replicates in which the protein was quantifiable. ^b^ The average log_2_ ratio of protein quantity representing (inoculated/mock) from three biological replicates. ^c^ The *p*-value was calculated by Student’s *t*-test if the protein could be quantified from three biological replicates. Color codes: red, significant quantity change with greater than 0.58 of log_2_ ratio (*p* < 0.05); blue, significant quantity change less than −0.58 of log_2_ ratio; grey, no significant change between the inoculated and mock group (*p* ≥ 0.05) or not quantified in all three replicates (NQ); white, the quantity change is between 0.58 and −0.58 of log_2_ ratio (*p* < 0.05).

**Table 5:** Proteins involved in energy metabolisms with a significant change in abundance at 24, 48 and 96 h post-inoculation with *P. infestans*.

			24 h			48 h			96 h		
Gene Accession	Protein Description	Arabidopsis Homolog	# rep ^a^	log_2_ ratio ^b^	*p* ^c^	# rep ^a^	log_2_ ratio ^b^	*p* ^c^	# rep ^a^	log_2_ ratio ^b^	*p* ^c^
**Metabolism-Primary-Energy Metabolisms**											
Solyc11g007720	pyruvate dehydrogenase complex (PDC)	AT3G52200	3	−0.121	0.358	3	0.082	0.842	3	0.645	0.017
Solyc01g073740	citrate synthase (CSY)	AT2G44350	3	−0.112	0.410	3	−0.198	0.283	3	0.639	0.017
Solyc07g052350	aconitate hydratase (ACO)	AT2G05710	3	0.019	0.872	3	0.110	0.544	3	0.708	0.047
Solyc12g005860	aconitate hydratase (ACO)	AT2G05710	3	−0.084	0.054	3	−0.100	0.605	3	0.968	0.023
Solyc01g005560	isocitrate dehydrogenase (IDH)	AT1G65930	3	−0.125	0.650	3	0.311	0.369	3	0.753	0.049
Solyc07g064800	oxoglutarate dehydrogenase complex (ODC)	AT4G26910	3	0.049	0.497	3	0.150	0.700	3	0.607	0.046
Solyc12g005080	oxoglutarate dehydrogenase complex (ODC)	AT5G55070	3	−0.073	0.661	3	0.107	0.043	3	1.003	0.023
Solyc06g083790	succinyl-CoA ligase (SCoAL)	AT2G20420	3	0.058	0.107	3	0.039	0.888	3	0.602	0.016
Solyc01g007910	succinyl-CoA ligase (SCoAL)	AT5G23250	3	−0.131	0.132	3	0.295	0.529	3	0.630	0.035
Solyc09g075450	fumarase (FUM)	AT2G47510	3	0.088	0.362	3	0.091	0.691	3	0.953	0.004
Solyc08g066360	malic enzyme (ME)	AT1G79750	3	−0.007	0.978	3	−0.016	0.957	3	0.903	0.045
**Metabolism-Primary-Glycolysis**											
Solyc07g045160	ATP-dependent 6-phosphofructokinase (PFK)	AT4G26270	3	0.217	0.106	3	0.107	0.497	3	1.052	0.037
Solyc10g005510	glyceraldehyde-3-phosphate dehydrogenase (GAPDH)	AT1G16300	3	0.097	0.413	3	0.220	0.713	3	1.096	0.045
Solyc03g114500	enolase (ENO1)	AT1G74030	3	0.599	0.049	3	−1.053	0.430	2	1.073	NQ
Solyc03g097680	pyruvate dehydrogenase 1 (PDH)	AT5G50850	3	−0.048	0.857	3	−0.270	0.724	3	1.251	0.038
**Metabolism-Primary-Carbohydrate Metabolisms-PPP**											
Solyc04g005160	6-phosphogluconate dehydrogenase (6PGD)	AT3G02360	3	−0.046	0.033	3	0.524	0.031	3	1.886	0.016
Solyc05g008370	ribose 5-phosphate isomerase A (Rpi)	AT1G71100	3	0.068	0.366	3	0.211	0.715	3	0.790	0.036
Solyc11g033288	transaldolase (TAL)	AT5G13420	3	0.029	0.751	3	−0.206	0.729	3	0.667	0.032
**Metabolism-Primary-Fatty Acid/Lipids**											
Solyc10g076600	acyl-CoA oxidase/dehydrogenase (ACAD)	AT3G51840	3	−0.057	0.872	3	0.050	0.928	3	2.085	0.014
Solyc01g059830	enoyl-CoA hydratase (ECH)	AT4G16210	3	0.469	0.386	2	−0.156	NQ	3	0.750	0.041
Solyc12g094450	enoyl-CoA hydratase (ECH)	AT1G76150	2	−0.278	NQ	3	0.202	0.216	3	1.663	0.002

^a^ Number of biological replicates in which the protein was quantifiable. ^b^ The average log_2_ ratio of protein quantity representing (inoculated/mock) from three biological replicates. ^c^ The *p*-value was calculated by Student’s *t*-test if the protein could be quantified from three biological replicates. Color codes: red, significant quantity change with greater than 0.58 of log_2_ ratio (*p* < 0.05); blue, significant quantity change less than −0.58 of log_2_ ratio; grey, no significant change between the inoculated and mock group (*p* ≥ 0.05) or not quantified in all three replicates (NQ); white, the quantity change is between 0.58 and −0.58 of log_2_ ratio (*p* < 0.05).

**Table 6:** Proteins involved in carbon fixation with a significant change in abundance at 24, 48 and 96 h post-inoculation with *P. infestans*.

			24 h			48 h			96 h		
Gene Accession	Protein Description	Arabidopsis Homolog	# rep ^a^	log_2_ ratio ^b^	*p* ^c^	# rep ^a^	log_2_ ratio ^b^	*p* ^c^	# rep ^a^	log_2_ ratio ^b^	*p* ^c^
Solyc06g063370	chlorophyll a-b binding protein (LHCB)	AT4G10340	3	−0.061	0.659	3	0.391	0.212	3	−0.662	0.014
Solyc12g005630	cytochrome b6-f complex iron-sulfur subunit	AT4G03280	3	‒0.368	0.077	3	‒0.001	0.998	3	‒0.739	0.001
Solyc06g009940	photosystem I (PSI) P700 chlorophyll a apoprotein	ATCG00350	3	0.029	0.810	3	‒0.121	0.869	3	‒0.836	0.015
Solyc02g069450	photosystem I (PSI) reaction center subunit III	AT1G31330	3	0.107	0.605	3	0.048	0.930	3	‒0.661	0.038
Solyc08g013670	photosystem I (PSI) reaction center subunit N	AT5G64040	3	0.416	0.244	3	0.412	0.498	3	‒0.854	0.033
Solyc05g150152	photosystem I (PSI) reaction centre subunit N protein	AT1G49975	2	0.073	NQ	3	‒0.279	0.587	3	‒0.599	0.048
Solyc06g060340	photosystem II (PSII) 22 kDa protein	AT1G44575	3	‒0.091	0.339	3	0.234	0.063	3	‒0.910	0.008
Solyc05g052600	sedoheptulose-1,7-bisphosphatase (SBPase)	AT3G55800	3	0.077	0.001	3	‒0.292	0.626	3	‒0.758	0.039

^a^ Number of biological replicates in which the protein was quantifiable. ^b^ The average log_2_ ratio of protein quantity representing (inoculated/mock) from three biological replicates. ^c^ The *p*-value was calculated by Student’s *t*-test if the protein could be quantified from three biological replicates. Color codes: red, significant quantity change with greater than 0.58 of log_2_ ratio (*p* < 0.05); blue, significant quantity change less than −0.58 of log_2_ ratio; grey, no significant change between the inoculated and mock group (*p* ≥ 0.05) or not quantified in all three replicates (NQ); white, the quantity change is between 0.58 and −0.58 of log_2_ ratio (*p* < 0.05).

**Table 7:** Proteins involved in secondary metabolism with a significant change in abundance at 24, 48 and 96 h post-inoculation with *P. infestans*.

			24 h			48 h			96 h		
Gene Accession	Protein Description	Arabidopsis Homolog	# rep ^a^	log_2_ ratio ^b^	*p* ^c^	# rep ^a^	log_2_ ratio ^b^	*p* ^c^	# rep ^a^	log_2_ ratio ^b^	*p* ^c^
Solyc06g150137	cinnamate 4-hydroxylase (C4H)	AT2G30490	3	−0.075	0.505	3	0.164	0.736	3	1.390	0.004
Solyc03g117870	4-coumarate:CoA ligase (4CL)	AT3G21240	3	0.185	0.366	3	0.622	0.178	3	2.293	0.004
Solyc01g091190	5-enolpyruvylshikimate-3-phosphate synthase (ESPS)	AT2G45300	3	−0.124	0.369	3	0.208	0.229	3	1.165	0.022

^a^ Number of biological replicates in which the protein was quantifiable. ^b^ The average log_2_ ratio of protein quantity representing (inoculated/mock) from three biological replicates. ^c^ The *p*-value was calculated by Student’s *t*-test if the protein could be quantified from three biological replicates. Color codes: red, significant quantity change with greater than 0.58 of log_2_ ratio (*p* < 0.05); blue, significant quantity change less than −0.58 of log_2_ ratio; grey, no significant change between the inoculated and mock group (*p* ≥ 0.05) or not quantified in all three replicates (NQ); white, the quantity change is between 0.58 and −0.58 of log_2_ ratio (*p* < 0.05).

## Data Availability

The mass spectrometry proteomics data have been deposited to the ProteomeXchange Consortium via the PRIDE partner repository (http://proteomecentral.proteomexchange.org, accessed on 30 October 2020) with the dataset identifier PXD022266.

## Supplementary Materials

Supplementary materials can be found at https://www.mdpi.com/article/10.3390/ijms22084174/s1. Supplemental Figure S1, the expression profile of the marker genes used to clarify *P. infestans* pathogenesis stage. Supplemental Table S1, list of quantified tomato protein with a significant change in abundance at 24, 48, or 96 hpi of *P. infestans* compared to mock. Supplemental Table S2, full list of quantified proteins and peptides in three biological replicates of the infected (24, 48 and 96 hpi) and mock (24, 48 and 96 hpt) experiments. Supplemental Table S3, list of primer sequences for RT-PCR analysis of the *P. infestans* marker genes. Supplemental Table S4, list of primer sequences for qRT-PCR analysis of the selected tomato genes.

## Abbreviations

ABA	abscisic acid
DIA	data-independent acquisition
DDA	data-dependent acquisition
ET	ethylene
ETI	effector-triggered immunity
ETS	effector-triggered susceptibility
HR	hypersensitive response
JA	jasmonic acid
LB	late blight
PAMP	pathogen-associated molecular pattern
PCD	programmed cell death
PTI	PAMP-triggered immunity
ROS	Reactive-oxidative species
SA	salicylic acid

## References

[1] Schoina C., Govers F. (2015). The Oomycete Phytophthora Infestans, the Irish Potato Famine Pathogen. Principles of Plant-Microbe Interactions.

[2] Nowicki M., Fooled M.R., Nowakowska M., Kozik E.U. (2012). Potato and Tomato Late Blight Caused by Phytophthora Infestans: An Overview of Pathology and Resistance Breeding. Plant Dis..

[3] Elsayed A.Y., da Silva D.J.H., Carneiro P.C.S., Mizubuti E.S.G. (2012). The Inheritance of Late Blight Resistance Derived from Solanum Habrochaites. Crop. Breed. Appl. Biot..

[4] Kamoun S., Smart C.D. (2005). Late Blight of Potato and Tomato in the Genomics Era. Plant Dis..

[5] Hausbeck M.K., Lamour K.H. (2004). Phytophthora Capsici on Vegetable Crops: Research Progress and Management Challenges. Plant Dis..

[6] Glazebrook J. (2005). Contrasting Mechanisms of Defense against Biotrophic and Necrotrophic Pathogens. Annu. Rev. Phytopathol..

[7] Panstruga R., Dodds P.N. (2009). Terrific Protein Traffic: The Mystery of Effector Protein Delivery by Filamentous Plant Pathogens. Science.

[8] Koeck M., Hardham A.R., Dodds P.N. (2011). The Role of Effectors of Biotrophic and Hemibiotrophic Fungi in Infection. Cellular Microbiol..

[9] Wang B.L., Liu J., Tian Z.D., Song B.T., Xie C.H. (2005). Monitoring the Expression Patterns of Potato Genes Associated with Quantitative Resistance to Late Blight During Phytophthora Infestans Infection Using Cdna Microarrays. Plant Sci..

[10] Cai G., Restrepo S., Myers K., Zuluaga P., Danies G., Smart C., Fry W. (2013). Gene Profiling in Partially Resistant and Susceptible near-Isogenic Tomatoes in Response to Late Blight in the Field. Mol. Plant Pathol..

[11] Zuluaga A.P., Vega-Arreguin J.C., Fei Z., Matas A.J., Patev S., Fry W.E., Rose J.K. (2016). Analysis of the Tomato Leaf Transcriptome During Successive Hemibiotrophic Stages of a Compatible Interaction with the Oomycete Pathogen Phytophthora Infestans. Mol. Plant Pathol..

[12] Larsen M.K., Jorgensen M.M., Bennike T.B., Stensballe A. (2016). Time-Course Investigation of Phytophthora Infestans Infection of Potato Leaf from Three Cultivars by Quantitative Proteomics. Data Brief..

[13] Ali A., Alexandersson E., Sandin M., Resjo S., Lenman M., Hedley P., Levander F., Andreasson E. (2014). Quantitative Proteomics and Transcriptomics of Potato in Response to Phytophthora Infestans in Compatible and Incompatible Interactions. BMC Genom..

[14] Xiao C., Gao J., Zhang Y., Wang Z., Zhang D., Chen Q., Ye X., Xu Y., Yang G., Yan L. (2019). Quantitative Proteomics of Potato Leaves Infected with Phytophthora Infestans Provides Insights into Coordinated and Altered Protein Expression During Early and Late Disease Stages. Int. J. Mol. Sci..

[15] Laurindo B., Laurindo R., Fontes P., Vital C., Delazari F., Baracat-Pereira M., da Silva D. (2018). Comparative Proteomics Reveals Set of Oxidative Stress and Thaumatin-Like Proteins Associated with Resistance to Late Blight of Tomato. Am. J. Plant Sci..

[16] Kelley B.S., Lee S.J., Damasceno C.M., Chakravarthy S., Kim B.D., Martin G.B., Rose J.K. (2010). A Secreted Effector Protein (Sne1) from Phytophthora Infestans Is a Broadly Acting Suppressor of Programmed Cell Death. Plant J..

[17] van West P., de Jong A.J., Judelson H.S., Emons A.M.C., Govers F. (1998). The Ipio Gene of Phytophthora Infestans Is Highly Expressed in Invading Hyphae During Infection. Fungal Genet. Biol..

[18] Kanneganti T.-D., Huitema E., Cakir C., Kamoun S. (2006). Synergistic Interactions of the Plant Cell Death Pathways Induced by Phytophthora Infestans Nep1-Like Protein Pinpp1. 1 and Inf1 Elicitin. Mol. Plant-Microbe Interact..

[19] Bartnicki-Garcia S. (1968). Cell Wall Chemistry, Morphogenesis, and Taxonomy of Fungi. Annu. Rev. Microbiol..

[20] Salzman R.A., Koiwa H., Ibeas J.I., Pardo J.M., Hasegawa P.M., Bressan R.A. (2004). Inorganic Cations Mediate Plant Pr5 Protein Antifungal Activity through Fungal Mnn1- and Mnn4-Regulated Cell Surface Glycans. Mol. Plant Microbe Interact..

[21] Batalia M.A., Monzingo A.F., Ernst S., Roberts W., Robertus J.D. (1996). The Crystal Structure of the Antifungal Protein Zeamatin, a Member of the Thaumatin-Like, PR-5 Protein Family. Nat. Struct. Biol..

[22] Ali S., Ganai B.A., Kamili A.N., Bhat A.A., Mir Z.A., Bhat J.A., Tyagi A., Islam S.T., Mushtaq M., Yadav P. (2018). Pathogenesis-Related Proteins and Peptides as Promising Tools for Engineering Plants with Multiple Stress Tolerance. Microbiol. Res..

[23] Bantignies B., Seguin J., Muzac I., Dedaldechamp F., Gulick P., Ibrahim R. (2000). Direct Evidence for Ribonucleolytic Activity of a PR-10-Like Protein from White Lupin Roots. Plant Mol. Biol..

[24] Zhou X.J., Lu S., Xu Y.H., Wang J.W., Chen X.Y. (2002). A Cotton Cdna (Gapr-10) Encoding a Pathogenesis-Related 10 Protein with in Vitro Ribonuclease Activity. Plant Sci..

[25] Andrade L.B., Oliveira A.S., Ribeiro J.K., Kiyota S., Vasconcelos I.M., de Oliveira J.T., de Sales M.P. (2010). Effects of a Novel Pathogenesis-Related Class 10 (PR-10) Protein from Crotalaria Pallida Roots with Papain Inhibitory Activity against Root-Knot Nematode Meloidogyne Incognita. J. Agric. Food Chem..

[26] Guevara-Morato M.A., de Lacoba M.G., Garcia-Luque I., Serra M.T. (2010). Characterization of a Pathogenesis-Related Protein 4 (PR-4) Induced in Capsicum Chinense L3 Plants with Dual Rnase and Dnase Activities. J. Exp. Bot..

[27] Klarzynski O., Plesse B., Joubert J.M., Yvin J.C., Kopp M., Kloareg B., Fritig B. (2000). Linear Beta-1,3 Glucans Are Elicitors of Defense Responses in Tobacco. Plant Physiol..

[28] Chen Y.L., Lee C.Y., Cheng K.T., Chang W.H., Huang R.N., Nam H.G., Chen Y.R. (2014). Quantitative Peptidomics Study Reveals That a Wound-Induced Peptide from PR-1 Regulates Immune Signaling in Tomato. Plant Cell.

[29] Chen Y.L., Fan K.T., Hung S.C., Chen Y.R. (2020). The Role of Peptides Cleaved from Protein Precursors in Eliciting Plant Stress Reactions. New Phytol..

[30] Gamir J., Darwiche R., Van’t Hof P., Choudhary V., Stumpe M., Schneiter R., Mauch F. (2017). The Sterol-Binding Activity of Pathogenesis-Related Protein 1 Reveals the Mode of Action of an Antimicrobial Protein. Plant J..

[31] Kruger J., Thomas C.M., Golstein C., Dixon M.S., Smoker M., Tang S.K., Mulder L., Jones J.D.G. (2002). A Tomato Cysteine Protease Required for Cf-2-Dependent Disease Resistance and Suppression of Autonecrosis. Science.

[32] Tian M., Win J., Song J., van der Hoorn R., van der Knaap E., Kamoun S. (2007). A Phytophthora Infestans Cystatin-Like Protein Targets a Novel Tomato Papain-Like Apoplastic Protease. Plant Physiol..

[33] Zhou L., Cheung M.-Y., Li M.-W., Fu Y., Sun Z., Sun S.-M., Lam H.-M. (2010). Rice Hypersensitive Induced Reaction Protein 1 (Oshir1) Associates with Plasma Membrane and Triggers Hypersensitive Cell Death. BMC Plant Biol..

[34] Qi Y., Tsuda K., Nguyen le V., Wang X., Lin J., Murphy A.S., Glazebrook J., Thordal-Christensen H., Katagiri F. (2011). Physical Association of Arabidopsis Hypersensitive Induced Reaction Proteins (Hirs) with the Immune Receptor Rps2. J. Biol. Chem..

[35] Lampl N., Alkan N., Davydov O., Fluhr R. (2013). Set-Point Control of Rd21 Protease Activity by Atserpin1 Controls Cell Death in Arabidopsis. Plant J..

[36] Liu Y., Wang K., Cheng Q., Kong D., Zhang X., Wang Z., Wang Q., Xie Q., Yan J., Chu J. (2020). Cysteine Protease Rd21a Regulated by E3 Ligase Sinat4 Is Required for Drought-Induced Resistance to Pseudomonas syringae in Arabidopsis. J. Exp. Bot..

[37] DiMario R.J., Clayton H., Mukherjee A., Ludwig M., Moroney J.V. (2017). Plant Carbonic Anhydrases: Structures, Locations, Evolution, and Physiological Roles. Mol. Plant.

[38] Zhou Y., Vroegop-Vos I.A., Van Dijken A.J.H., Van der Does D., Zipfel C., Pieterse C.M.J., Van Wees S.C.M. (2020). Carbonic Anhydrases Ca1 and Ca4 Function in Atmospheric Co2-Modulated Disease Resistance. Planta.

[39] Slaymaker D.H., Navarre D.A., Clark D., del Pozo O., Martin G.B., Klessig D.F. (2002). The Tobacco Salicylic Acid-Binding Protein 3 (Sabp3) Is the Chloroplast Carbonic Anhydrase, Which Exhibits Antioxidant Activity and Plays a Role in the Hypersensitive Defense Response. Proc. Natl. Acad. Sci. USA.

[40] Restrepo S., Myers K.L., del Pozo O., Martin G.B., Hart A.L., Buell C.R., Fry W.E., Smart C.D. (2005). Gene Profiling of a Compatible Interaction between Phytophthora Infestans and Solanum Tuberosum Suggests a Role for Carbonic Anhydrase. Mol. Plant-Microbe Interact..

[41] Wirthmueller L., Zhang Y., Jones J.D., Parker J.E. (2007). Nuclear Accumulation of the Arabidopsis Immune Receptor Rps4 Is Necessary for Triggering Eds1-Dependent Defense. Curr. Biol..

[42] Liu Y., Schiff M., Marathe R., Dinesh-Kumar S.P. (2002). Tobacco Rar1, Eds1 and NPR1/Nim1 Like Genes Are Required for N-Mediated Resistance to Tobacco Mosaic Virus. Plant J..

[43] Hu G.S., deHart A.K.A., Li Y.S., Ustach C., Handley V., Navarre R., Hwang C.F., Aegerter B.J., Williamson V.M., Baker B. (2005). Eds1 in Tomato Is Required for Resistance Mediated by Tir-Class R Genes and the Receptor-Like R Gene Ve. Plant J..

[44] Parker J.E., Holub E.B., Frost L.N., Falk A., Gunn N.D., Daniels M.J. (1996). Characterization of Eds1, a Mutation in Arabidopsis Suppressing Resistance to Peronospora Parasitica Specified by Several Different Rpp Genes. Plant Cell.

[45] Heidrich K., Wirthmueller L., Tasset C., Pouzet C., Deslandes L., Parker J.E. (2011). Arabidopsis Eds1 Connects Pathogen Effector Recognition to Cell Compartment-Specific Immune Responses. Science.

[46] Glazebrook J., Rogers E.E., Ausubel F.M. (1996). Isolation of Arabidopsis Mutants with Enhanced Disease Susceptibility by Direct Screening. Genetics.

[47] Cui H., Gobbato E., Kracher B., Qiu J., Bautor J., Parker J.E. (2017). A Core Function of Eds1 with Pad4 Is to Protect the Salicylic Acid Defense Sector in Arabidopsis Immunity. New Phytol..

[48] Li J., Brader G., Palva E.T. (2008). Kunitz Trypsin Inhibitor: An Antagonist of Cell Death Triggered by Phytopathogens and Fumonisin B1 in Arabidopsis. Mol. Plant.

[49] Arnaiz A., Talavera-Mateo L., Gonzalez-Melendi P., Martinez M., Diaz I., Santamaria M.E. (2018). Arabidopsis Kunitz Trypsin Inhibitors in Defense against Spider Mites. Front. Plant Sci..

[50] Heitz T., Bergey D.R., Ryan C.A. (1997). A Gene Encoding a Chloroplast-Targeted Lipoxygenase in Tomato Leaves Is Transiently Induced by Wounding, Systemin, and Methyl Jasmonate. Plant Physiol..

[51] Zhao Y., Thilmony R., Bender C.L., Schaller A., He S.Y., Howe G.A. (2003). Virulence Systems of Pseudomonas syringae pv. Tomato Promote Bacterial Speck Disease in Tomato by Targeting the Jasmonate Signaling Pathway. Plant J..

[52] Pauwels L., Barbero G.F., Geerinck J., Tilleman S., Grunewald W., Perez A.C., Chico J.M., Vanden Bossche R., Sewell J., Gil E. (2010). Ninja Connects the Co-Repressor Topless to Jasmonate Signalling. Nature.

[53] Causier B., Ashworth M., Guo W., Davies B. (2012). The Topless Interactome: A Framework for Gene Repression in Arabidopsis. Plant Physiol..

[54] Harvey S., Kumari P., Lapin D., Griebel T., Hickman R., Guo W., Zhang R., Parker J.E., Beynon J., Denby K. (2020). Downy Mildew Effector Harxl21 Interacts with the Transcriptional Repressor Topless to Promote Pathogen Susceptibility. bioRxiv.

[55] Mochizuki N., Brusslan J.A., Larkin R., Nagatani A., Chory J. (2001). Arabidopsis Genomes Uncoupled 5 (Gun5) Mutant Reveals the Involvement of Mg-Chelatase H Subunit in Plastid-to-Nucleus Signal Transduction. Proc. Natl. Acad. Sci. USA.

[56] Ibata H., Nagatani A., Mochizuki N. (2016). Chlh/Gun5 Function in Tetrapyrrole Metabolism Is Correlated with Plastid Signaling but Not Aba Responses in Guard Cells. Front. Plant Sci..

[57] Shang Y., Yan L., Liu Z.Q., Cao Z., Mei C., Xin Q., Wu F.Q., Wang X.F., Du S.Y., Jiang T. (2010). The Mg-Chelatase H Subunit of Arabidopsis Antagonizes a Group of Wrky Transcription Repressors to Relieve Aba-Responsive Genes of Inhibition. Plant Cell.

[58] Berens M.L., Berry H.M., Mine A., Argueso C.T., Tsuda K. (2017). Evolution of Hormone Signaling Networks in Plant Defense. Annu. Rev. Phytopathol..

[59] Sagi M., Davydov O., Orazova S., Yesbergenova Z., Ophir R., Stratmann J.W., Fluhr R. (2004). Plant Respiratory Burst Oxidase Homologs Impinge on Wound Responsiveness and Development in Lycopersicon Esculentum. Plant Cell.

[60] Kumar S., Kaur A., Chattopadhyay B., Bachhawat A.K. (2015). Defining the Cytosolic Pathway of Glutathione Degradation in Arabidopsis Thaliana: Role of the Chac/Gcg Family of Gamma-Glutamyl Cyclotransferases as Glutathione-Degrading Enzymes and Atlap1 as the Cys-Gly Peptidase. Biochem. J..

[61] Rojas C.M., Senthil-Kumar M., Tzin V., Mysore K. (2014). Regulation of Primary Plant Metabolism during Plant-Pathogen Interactions and Its Contribution to Plant Defense. Front. Plant Sci..

[62] Dixon R.A., Achnine L., Kota P., Liu C.J., Reddy M.S., Wang L. (2002). The Phenylpropanoid Pathway and Plant Defence—A Genomics Perspective. Mol. Plant Pathol..

[63] Vogt T. (2010). Phenylpropanoid Biosynthesis. Mol. Plant.

[64] McManus J., Cheng Z., Vogel C. (2015). Next-Generation Analysis of Gene Expression Regulation--Comparing the Roles of Synthesis and Degradation. Mol. Biosyst..

[65] Liu Y., Beyer A., Aebersold R. (2016). On the Dependency of Cellular Protein Levels on Mrna Abundance. Cell.

[66] Tsuzuki T., Takahashi K., Inoue S., Okigaki Y., Tomiyama M., Hossain M.A., Shimazaki K., Murata Y., Kinoshita T. (2011). Mg-Chelatase H Subunit Affects Aba Signaling in Stomatal Guard Cells, but Is Not an Aba Receptor in Arabidopsis Thaliana. J. Plant Res..

[67] Zhang D.-P., Wu Z.-Y., Li X.-Y., Zhao Z.-X. (2002). Purification and Identification of a 42-Kilodalton Abscisic Acid-Specific-Binding Protein from Epidermis of Broad Bean Leaves. Plant Physiol..

[68] Wu F.Q., Xin Q., Cao Z., Liu Z.Q., Du S.Y., Mei C., Zhao C.X., Wang X.F., Shang Y., Jiang T. (2009). The Magnesium-Chelatase H Subunit Binds Abscisic Acid and Functions in Abscisic Acid Signaling: New Evidence in Arabidopsis. Plant Physiol..

[69] Shen Y.Y., Wang X.F., Wu F.Q., Du S.Y., Cao Z., Shang Y., Wang X.L., Peng C.C., Yu X.C., Zhu S.Y. (2006). The Mg-Chelatase H Subunit Is an Abscisic Acid Receptor. Nature.

[70] Muller A.H., Hansson M. (2009). The Barley Magnesium Chelatase 150-Kd Subunit Is Not an Abscisic Acid Receptor. Plant Physiol..

[71] Chan K.X., Phua S.Y., Crisp P., McQuinn R., Pogson B.J. (2016). Learning the Languages of the Chloroplast: Retrograde Signaling and Beyond. Annu. Rev. Plant Biol..

[72] Agrios G.N. (2005). Plant Pathology.

[73] Li Y., Han Y., Qu M., Chen J., Chen X., Geng X., Wang Z., Chen S. (2020). Apoplastic Cell Death-Inducing Proteins of Filamentous Plant Pathogens: Roles in Plant-Pathogen Interactions. Front. Genet..

[74] Caten C., Jinks J. (1968). Spontaneous Variability of Single Isolates of Phytophthora Infestans. I. Cultural Variation. Can. J. Bot..

[75] Wilson U., Coffey M. (1980). Cytological Evaluation of General Resistance to Phytophthora Infestans in Potato Foliage. Ann. Bot..

[76] Yin J., Gu B., Huang G., Tian Y., Quan J., Lindqvist-Kreuze H., Shan W. (2017). Conserved Rxlr Effector Genes of Phytophthora Infestans Expressed at the Early Stage of Potato Infection Are Suppressive to Host Defense. Front. Plant Sci..

[77] Schneider C.A., Rasband W.S., Eliceiri K.W. (2012). Nih Image to Imagej: 25 Years of Image Analysis. Nat. Methods.

[78] Faurobert M., Pelpoir E., Chaib J. (2007). Phenol Extraction of Proteins for Proteomic Studies of Recalcitrant Plant Tissues. Methods Mol. Biol..

[79] Wu X., Xiong E., Wang W., Scali M., Cresti M. (2014). Universal Sample Preparation Method Integrating Trichloroacetic Acid/Acetone Precipitation with Phenol Extraction for Crop Proteomic Analysis. Nat. Protoc..

[80] Saravanan R.S., Rose J.K. (2004). A Critical Evaluation of Sample Extraction Techniques for Enhanced Proteomic Analysis of Recalcitrant Plant Tissues. Proteomics.

[81] Fan K.T., Wang K.H., Chang W.H., Yang J.C., Yeh C.F., Cheng K.T., Hung S.C., Chen Y.R. (2019). Application of Data-Independent Acquisition Approach to Study the Proteome Change from Early to Later Phases of Tomato Pathogenesis Responses. Int. J. Mol. Sci..

[82] Chen C.J., Chen W.Y., Tseng M.C., Chen Y.R. (2012). Tunnel Frit: A Nonmetallic in-Capillary Frit for Nanoflow Ultra High-Performance Liquid Chromatography-Mass Spectrometryapplications. Anal. Chem..

[83] Scheltema R.A., Hauschild J.P., Lange O., Hornburg D., Denisov E., Damoc E., Kuehn A., Makarov A., Mann M. (2014). The Q Exactive HF, a Benchtop Mass Spectrometer with a Pre-Filter, High-Performance Quadrupole and an Ultra-High-Field Orbitrap Analyzer. Mol. Cell. Proteom. MCP.

[84] Bruderer R., Bernhardt O.M., Gandhi T., Xuan Y., Sondermann J., Schmidt M., Gomez-Varela D., Reiter L. (2017). Optimization of Experimental Parameters in Data-Independent Mass Spectrometry Significantly Increases Depth and Reproducibility of Results. Mol. Cell. Proteom. MCP.

[85] Craig R., Beavis R.C. (2004). Tandem: Matching Proteins with Tandem Mass Spectra. Bioinformatics.

[86] Keller A., Nesvizhskii A.I., Kolker E., Aebersold R. (2002). Empirical Statistical Model to Estimate the Accuracy of Peptide Identifications Made by MS/MS and Database Search. Anal. Chem..

[87] Shteynberg D., Deutsch E.W., Lam H., Eng J.K., Sun Z., Tasman N., Mendoza L., Moritz R.L., Aebersold R., Nesvizhskii A.I. (2011). Iprophet: Multi-Level Integrative Analysis of Shotgun Proteomic Data Improves Peptide and Protein Identification Rates and Error Estimates. Mol. Cell. Proteom. MCP.

[88] Deutsch E.W., Mendoza L., Shteynberg D., Slagel J., Sun Z., Moritz R.L. (2015). Trans-Proteomic Pipeline, a Standardized Data Processing Pipeline for Large-Scale Reproducible Proteomics Informatics. Proteom. Clin. Appl..

[89] Reiter L., Claassen M., Schrimpf S.P., Jovanovic M., Schmidt A., Buhmann J.M., Hengartner M.O., Aebersold R. (2009). Protein Identification False Discovery Rates for Very Large Proteomics Data Sets Generated by Tandem Mass Spectrometry. Mol. Cell. Proteom. MCP.

[90] Lam H., Deutsch E.W., Eddes J.S., Eng J.K., Stein S.E., Aebersold R. (2008). Building Consensus Spectral Libraries for Peptide Identification in Proteomics. Nat. Methods.

[91] Röst H.L., Sachsenberg T., Aiche S., Bielow C., Weisser H., Aicheler F., Andreotti S., Ehrlich H.C., Gutenbrunner P., Kenar E. (2016). OpenMS: A Flexible Open-Source Software Platform for Mass Spectrometry Data Analysis. Nat. Methods.

[92] Schubert O.T., Gillet L.C., Collins B.C., Navarro P., Rosenberger G., Wolski W.E., Lam H., Amodei D., Mallick P., MacLean B. (2015). Building High-Quality Assay Libraries for Targeted Analysis of Swath MS Data. Nat. Protoc..

[93] Röst H.L., Rosenberger G., Navarro P., Gillet L., Miladinović S.M., Schubert O.T., Wolski W., Collins B.C., Malmström J., Malmström L. (2014). OpenSWATH Enables Automated, Targeted Analysis of Data- Independent Acquisition MS Data. Nat. Biotechnol..

[94] Lambert J.P., Ivosev G., Couzens A.L., Larsen B., Taipale M., Lin Z.Y., Zhong Q., Lindquist S., Vidal M., Aebersold R. (2013). Mapping Differential Interactomes by Affinity Purification Coupled with Data-Independent Mass Spectrometry Acquisition. Nat. Methods.

[95] Perez-Riverol Y., Csordas A., Bai J., Bernal-Llinares M., Hewapathirana S., Kundu D.J., Inuganti A., Griss J., Mayer G., Eisenacher M. (2019). The PRIDE Database and Related Tools and Resources in 2019: Improving Support for Quantification Data. Nucleic Acids Res..

