# Supplementary figures and images for: Quantitative Proteomics Reveals the Dynamic Regulation of the Tomato Proteome in Response to Phytophthora infestans
Source: Int J Mol Sci. 2021 Apr 17;22(8):4174. doi: 10.3390/ijms22084174 (PMC8073981; doi:10.3390/ijms22084174)

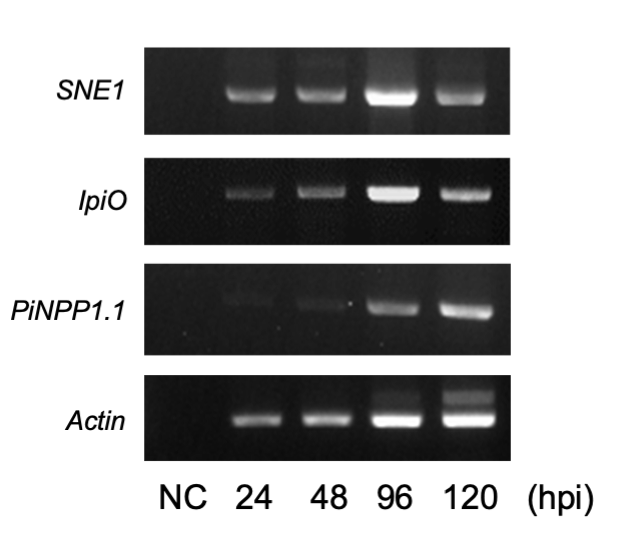

Supplement: Supplementary file 1 [file ijms-22-04174-s001.zip › figure S1.tiff]
